# DIMOND: DIffusion Model OptimizatioN with Deep Learning

**DOI:** 10.1002/advs.202307965

**Published:** 2024-04-18

**Authors:** Zihan Li, Ziyu Li, Berkin Bilgic, Hong‐Hsi Lee, Kui Ying, Susie Y. Huang, Hongen Liao, Qiyuan Tian

**Affiliations:** ^1^ School of Biomedical Engineering Tsinghua University Beijing 100084 P. R. China; ^2^ Wellcome Centre for Integrative Neuroimaging, FMRIB, Nuffield Department of Clinical Neurosciences University of Oxford Oxford OX3 9DU UK; ^3^ Athinoula A. Martinos Center for Biomedical Imaging Massachusetts General Hospital Charlestown MA 02129 USA; ^4^ Harvard Medical School Boston MA 02129 USA; ^5^ Department of Engineering Physics Tsinghua University Beijing 100084 P. R. China

**Keywords:** diffusion MRI, non‐linear optimization, microstructure imaging, self‐supervised learning

## Abstract

Diffusion magnetic resonance imaging is an important tool for mapping tissue microstructure and structural connectivity non‐invasively in the in vivo human brain. Numerous diffusion signal models are proposed to quantify microstructural properties. Nonetheless, accurate estimation of model parameters is computationally expensive and impeded by image noise. Supervised deep learning‐based estimation approaches exhibit efficiency and superior performance but require additional training data and may be not generalizable. A new DIffusion Model OptimizatioN framework using physics‐informed and self‐supervised Deep learning entitled “DIMOND” is proposed to address this problem. DIMOND employs a neural network to map input image data to model parameters and optimizes the network by minimizing the difference between the input acquired data and synthetic data generated via the diffusion model parametrized by network outputs. DIMOND produces accurate diffusion tensor imaging results and is generalizable across subjects and datasets. Moreover, DIMOND outperforms conventional methods for fitting sophisticated microstructural models including the kurtosis and NODDI model. Importantly, DIMOND reduces NODDI model fitting time from hours to minutes, or seconds by leveraging transfer learning. In summary, the self‐supervised manner, high efficacy, and efficiency of DIMOND increase the practical feasibility and adoption of microstructure and connectivity mapping in clinical and neuroscientific applications.

## Graphical abstract

This study proposes a self‐supervised and physics‐informed deep learning framework entitled DIMOND to accelerate and improve the diffusion MRI microstructural model fitting. This work systematically and quantitatively evaluates DIMOND on DTI, DKI and NODDI. DIMOND's superior performance, self‐supervised manner, and easy implementation and deployment increase the practical feasibility and adoption of microstructure and connectivity mapping in clinical and neuroscientific applications.

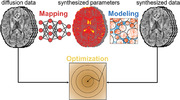

## 1 Introduction

Diffusion magnetic resonance imaging (MRI) is a useful tool for mapping the brain tissue microstructure non‐invasively at the cellular scale of micrometers. It is widely adopted in neuroscientific and clinical applications. In order to quantify a variety of microstructural properties at each image voxel, numerous biophysical models have been proposed to represent or model directional diffusion signals, such as the diffusion tensor model^[^ ^1^, ^2^, ^3^ ^]^ and multi‐tensor models (e.g., constrained spherical deconvolution^[^ ^4^, ^5^ ^]^ and BedpostX^[^ ^6^, ^7^, ^8^, ^9^ ^]^) for mapping axon orientation, diffusion kurtosis model for quantifying diffusion non‐Gaussianity,^[^ ^10^ ^]^ AxCaliber,^[^ ^11^ ^]^ ActiveAx,^[^ ^12^ ^]^ and TractCaliber^[^ ^13^ ^]^ for mapping axon diameter, NODDI (neurite orientation and dispersion density imaging)^[^ ^14^ ^]^ and SANDI^[^ ^15^ ^]^ for mapping neurite and soma density, and isotropic and anisotropic Kärger model^[^ ^16^ ^]^ (e.g., SMEX^[^ ^17^ ^]^ or NEXI^[^ ^18^ ^]^) for mapping water exchange between intra‐ and extra‐cellular spaces.

Nonetheless, fitting the microstructural model to diffusion data requires non‐linear optimization (e.g., Gauss‐Newton non‐linear optimization^[^ ^12^, ^14^ ^]^ and Markov Chain Monte Carlo sampling^[^ ^6^, ^12^ ^]^), which is often iterative and computationally expensive and has to be performed for millions of image voxels for each subject. The fitting process takes hours per subject depending on the model complexity, imaging spatial resolution and computing hardware, which imposes a major practical challenge for clinical applications that requires real‐time processing and visualization on the console computer and large‐scale imaging studies (e.g., HCP,^[^ ^19^ ^]^ UKB,^[^ ^20^, ^21^ ^]^ ADNI,^[^ ^22^ ^]^ and PPMI^[^ ^23^ ^]^) with massive datasets from hundreds or thousands of subjects. Novel optimization algorithms (e.g., Accelerated Microstructure Imaging via Convex Optimization (AMICO))^[^ ^24^ ^]^ and deployment (e.g., parallelizing computation using GPUs)^[^ ^25^, ^26^ ^]^ have been proposed to accelerate diffusion model fitting. Moreover, non‐linear optimization results heavily rely on the implementation, such as the optimization algorithm, noise assumption and initialization strategy, which is therefore often specifically designed for each diffusion model, slowing down the development, deployment, and distribution of new models. The Dmipy (Diffusion Microstructure Imaging in Python) toolbox is a representative effort that allows on‐the‐fly implementation, modeling, and optimization of any multi‐compartment diffusion models.^[^ ^27^ ^]^

Deep learning techniques, particularly neural networks (NNs), have demonstrated superior performance in accelerating and improving the estimation of diffusion model parameters.^[^ ^28^, ^29^, ^30^, ^31^, ^32^ ^]^ Previous works proposed to train NNs to map diffusion data to high‐quality reference parameter values using multilayer perceptrons^[^ ^28^, ^29^, ^33^, ^34^, ^35^, ^36^, ^37^ ^]^ or convolutional neural networks (CNNs).^[^ ^30^, ^31^, ^32^, ^38^, ^39^ ^]^ It only takes seconds to infer the results once NNs are trained. NNs also dramatically reduce the required diffusion‐weighted images (DWIs) and imaging time while maintaining the high quality of synthesis results, outperforming conventional model fitting methods.^[^ ^29^, ^30^ ^]^ For example, NNs have been demonstrated to generate accurate DTI results from six DWIs,^[^ ^40^ ^]^ radial kurtosis maps from 25 DWIs, and fiber orientation distribution functions from 20 DWIs.^[^ ^30^ ^]^ Moreover, a seminal supervised deep learning method for DTI based on the Recurrent Inference Machines (dtiRIM) not only generated high‐quality results but also successfully generalized to variations in the acquisition settings.^[^ ^41^ ^]^ As a result, advanced data‐intensive diffusion microstructural imaging methods become more practically feasible.

Nevertheless, current deep learning methods are mostly supervised, which induces several challenges. First, NNs trained on a specific dataset might be not optimal for another diffusion dataset acquired with different hardware systems, spatial resolutions, b‐values etc. It is particularly not trivial to generalize NNs across datasets acquired with different diffusion‐encoding directions since the image contrast is different.^[^ ^42^ ^]^ Second, the training targets are often obtained from remarkably longer scans on numerous subjects, which are difficult to acquire due to subject discomfort, motion artifacts and so on especially from patients, children and elderly subjects who cannot stay still for a long time. Third, generating diffusion model parameters using conventional methods as the training target introduces non‐negligible time cost.

To address these challenges, we propose a self‐supervised framework entitled “DIffusion Model OptimizatioN with Deep learning” (DIMOND) for fitting diffusion models to estimate microstructural properties. Specifically, DIMOND employs an NN to map noisy input diffusion data to model parameter values and optimizes NN's parameters by minimizing the difference between the input data and the synthetic data, which are generated via the forward model with the NN predictions, using gradient descent. Since the training can be performed on the data of each individual subject rather than from an external dataset to achieve self‐supervision (i.e., subject‐specific training), DIMOND does not suffer from the generalization problem commonly associated with supervised learning‐based methods. We first overview the framework of DIMOND and optimize NN architecture via solving the widely adopted diffusion tensor model non‐linearly using DIMOND on simulated data and empirical data provided by the Human Connectome Project (HCP). We further show that DIMOND's NN trained on HCP data can directly apply to data from Massachusetts General Hospital (MGH) Connectome Diffusion Microstructure Dataset (CDMD) to generate high‐quality results. Fine‐tuning NN parameters on the data of each CDMD subject in a self‐supervised manner for a few epochs further improves NN inferences, which are highly similar to those from subject‐specific training. Finally, we demonstrate the efficacy and efficiency of DIMOND for fitting more complex diffusion models that are solved using image intensity similarity as the optimization target, including the diffusion kurtosis model and the microstructural model in NODDI. Because of the superior performance, self‐supervised manner, and easy implementation and deployment, DIMOND has the potential to render diffusion microstructural imaging and modeling using deep learning more practically feasible and accessible, and improve upon traditional diffusion model fitting approaches.

## 2 DIMOND Methodology

### 2.1 DIMOND Framework

**Figure 1:**
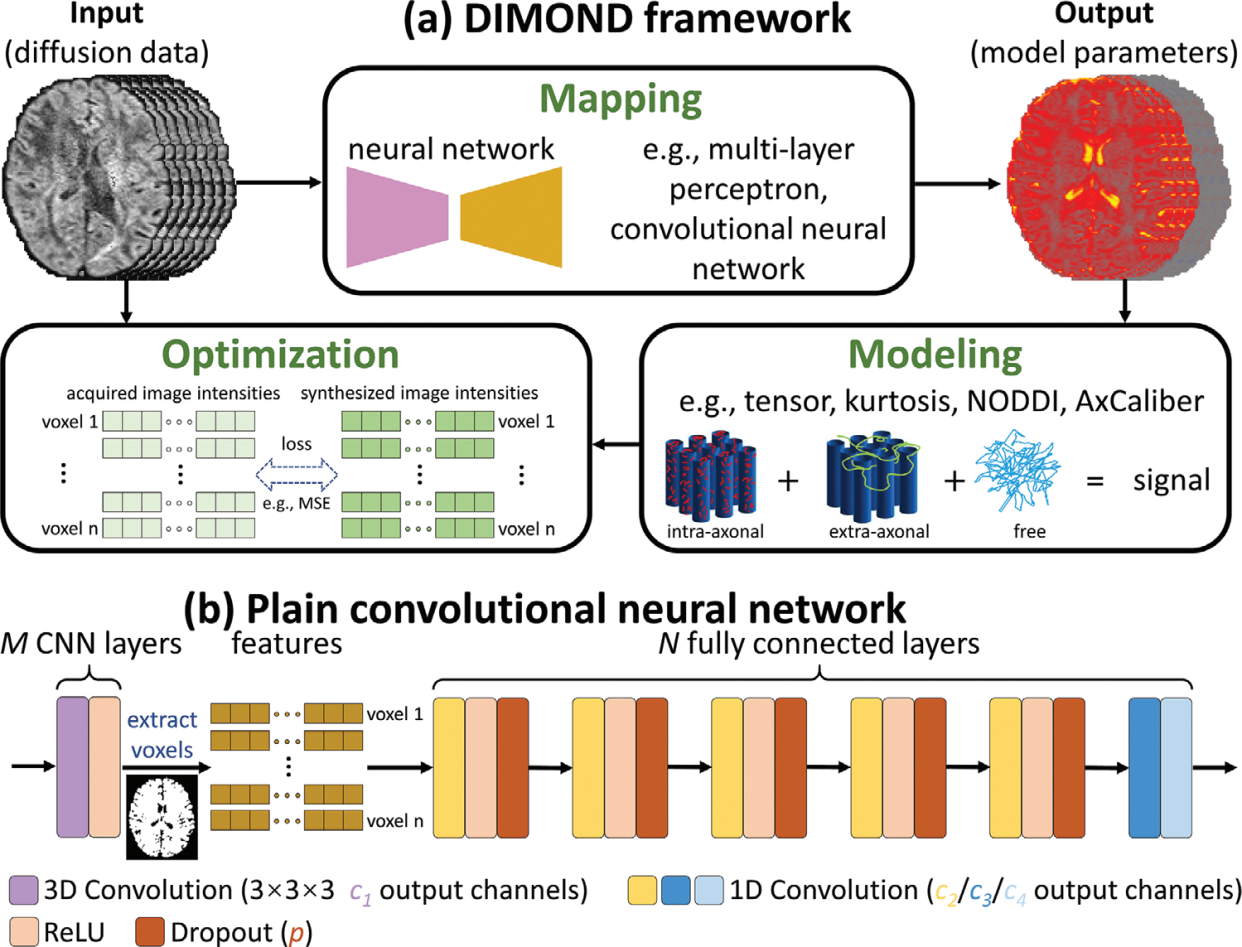
DIMOND framework. a) DIMOND consists of three steps, namely mapping, modeling and optimization. For the mapping, a neural network (NN) is utilized to transform input diffusion MRI data to unknown parameters of a diffusion model (e.g., one b = 0 image and six component maps for the diffusion tensor model). NN‐estimated parameters are then used to synthesize the input diffusion data via the forward model (e.g., tensor model) during the modeling process. During the optimization, the training of the NN aims to minimize the difference (e.g., mean squared error, MSE) between the acquired and synthesized diffusion data using gradient descent. Only the loss within the mask specified by the diffusion model assumption is considered. b) In this work, a plain NN is adopted, which consists of *M* 3 × 3 × 3 convolutional layers to utilize spatial redundancy and *N* fully connected layers. Each convolutional layer is paired with a ReLU activation layer. Each fully connected layer, except for the output layer, is paired with a ReLU activation layer and a dropout layer with a dropout rate of *p*.

DIMOND framework consists of three steps, namely mapping, modeling and optimization (**Figure** **1**a). Intuitively, DIMOND iteratively estimates diffusion model parameters from acquired diffusion signals using an NN during the mapping process, synthesizes input diffusion MRI data via the diffusion model parametrized by the network outputs during the modeling process, and approaches the optimal network parameters by minimizing the difference between the acquired and synthesized signals during the optimization process. Specifically, DIMOND employs an NN *G* to map input diffusion data ***I *** = [***I***_1_ … ***I***_***n***_]^*T*^  (where *n* is the number of image volumes) to parameter maps of a diffusion model *G*(***I***) = ***P *** = [***P***_1_ … ***P***_***m***_]^*T*^  (where *m* is the number of microstructural map volumes). The parameter maps are subsequently used to synthesize image volumes along identical diffusion encoding directions with identical b‐values as the input diffusion data via the forward model, resulting in $\hat{I}={\left[{\hat{I}}_{1}\cdots \;{\hat{I}}_{n}\right]}^{T}$. DIMOND's NN is optimized by minimizing the difference (e.g., mean squared error) between the raw acquired and synthesized image intensities (***I*** and $\hat{I}$) using gradient descent within the mask where the diffusion model parameters are of interest. Constraints that leverage prior knowledge of the diffusion model (e.g., noise distribution,^[^ ^43^ ^]^ sparsity,^[^ ^44^ ^]^ low‐rankness^[^ ^45^ ^]^) can be also incorporated into the loss function to further boost the performance. The modeling and optimization components of DIMOND vary across diffusion models and machine learning frameworks, which are therefore elaborated in the Experimental Section.

Although any NN can be used in the DIMOND framework, several requirements need to be considered. First, the number of network hyperparameters should be conservative. A lightweight NN reduces the computational load in the training and inference for shortened parameter estimation time especially on low‐cost accessible hardware systems, as well as to avoid overfitting given the limited training data from each individual subject for self‐supervision. Second, the NN should use convolution to utilize spatial similarity for denoising estimated parameter maps. Third, the generalizability should be sufficient. Fourth, synergistic techniques such as dropout can be used to avoid overfitting and improve performance. Finally, the last layer of the NN should produce estimates within ranges specified by a particular diffusion model (e.g., axonal volume fraction ranging between 0 and 1).

### 2.2 Network Deployment

In this study, a simple convolutional NN with a shallow and plain architecture and dropout layers which fulfills aforementioned requirements was adopted. Specifically, the NN consists of *M* layers of 3D convolution (size: 3 × 3 × 3, stride: 1 × 1 × 1) to utilize spatial information from all three dimensions followed by *N* layers of 1D convolution (size: 1 × 1 × 1, stride: 1 × 1 × 1) to integrate features along channels. When *M* = 0, DIMOND generates model parameters of each voxel solely using diffusion signals of the voxel itself. If *M* > 0, DIMOND utilizes information of neighboring voxels for the estimation of the central voxel's parameter. The receptive view corresponds to 2 × *M* + 1 voxels. *M* + *N* was fixed to seven to keep the NN shallow in this study. Specifically, the set of M = 0 and N = 7 was used for the simulation data while the set of *M* = 1 and *N* = 6 was used for the empirical data to capitalize spatial redundancy. Output channel numbers of hidden layers were fixed to *c_2_* = 256 and *c_3_* = 64. Output channel numbers of the first and last layer (i.e., *c_1_* and *c_4_*) were determined by the diffusion model. *c_4_* is equal to the number of unknown parameters of the diffusion model to estimate.

Each 1D convolution layer with *c_2_* output channels is paired with a dropout layer, which randomly discards features according to the dropout rate *p* during the training to avoid overfitting. *p* is fixed to 0.05 in this study. The dropout layer is also active during the inference to perform Monte Carlo (MC) dropout.^[^ ^46^, ^47^ ^]^ The trained NN is applied *l* times to generate *l* inferences, with each inference exhibiting slight variations from others since the network slightly varies with a positive dropout rate. Averaging *l* inferences (denoted by DIMOND‐MC*l*) reduces the variance of each individual inference (denoted by DIMOND‐MC1) and ultimately improves performance. The standard deviation of the *l* inferences at each voxel measures the network estimation uncertainty. In our experiments, *l* is fixed at 20.

## 3 Experimental Section

### 3.1 Data

#### 3.1.1 Simulation Data

Synthetic diffusion signals were generated to evaluate the efficacy of DIMOND. Specifically, a total of 64 × 64 × 128 voxels were simulated using the multi‐tensor model implemented in DIPY.^[^ ^48^ ^]^ The eigenvalues of each tensor were set to [0.0015, 0.0003, 0.0003] mm^2^ s^−1^.^[^ ^49^ ^]^ The number of tensors was randomly set to 1, 2, or 3. The spherical coordinate (*θ*, *Φ*) of the principal axis of each tensor was randomly selected from 0° to 360° and ‐90° to 90° respectively.

**Figure 2:**
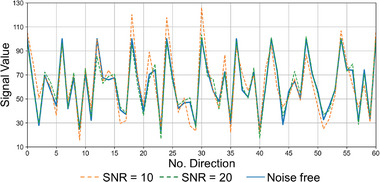
Simulation data. The first 61 diffusion signals of a representative voxel simulated without noise (blue solid line) and at a signal‐to‐noise ratio (SNR) of 10 (orange dashed line) and 20 (green dashed line) are plotted.

At each voxel, 236 data samples were generated at b = 0 and b = 1000 s mm^−2^. The b‐values and b‐vectors of the first 108 signals were identical to those from the HCP data, including 18 b = 0 and 90 b = 1000 s mm^−2^ diffusion‐weighted signals. The rest 128 diffusion‐weighted signals were simulated at b = 1000 s mm^−2^ along diffusion‐encoding directions uniformly distributed over a unit sphere. The simulated signals were generated without noise (i.e., ground truth) and with Rician noise at a signal‐to‐noise ratio (SNR) of 10 and 20. Exemplary 61 simulated signal values from a representative voxel were shown in **Figure** **2**.

The first 1/1/1/2/3/4/5 signals at b = 0 and the first 6/8/10/20/30/40/50 diffusion‐weighted signals at b = 1000 s mm^−2^ were used as input signals for fitting the widely adopted diffusion tensor model to evaluate the performance of DIMOND. The diffusion‐encoding directions of the HCP data were designed such that any first *N* directions are uniformly distributed over a unit sphere.

#### 3.1.2 HCP Diffusion Data

Pre‐processed diffusion MRI data acquired at 1.25 mm isotropic spatial resolution of 10 unrelated healthy subjects from the HCP WU‐Minn‐Ox Consortium were used. The data of each subject consisted of 18 b = 0 image volumes and 90 DWI volumes at b = 1000, 2000, and 3000 s mm^−2^, respectively. T_1_‐weighted (T1w) data and the mask of the entire brain of these subjects were also used. The acquisition protocols and pre‐processing pipelines have been described in detail previously.^[^ ^19^, ^50^, ^51^ ^]^

To evaluate the performance of DIMOND for different diffusion models, namely the diffusion tensor model, the diffusion kurtosis model and the NODDI model, three datasets representing 6‐fold acceleration in data acquisition were created by sub‐sampling the q‐space data for each HCP subject:

- HCP‐1Shell: Single‐shell data consisting of all 18 b = 0 image volumes and 90 DWI volumes at b = 1000 s mm^−2^ were used for computing reference tensor parameters and DTI metrics. A subset of the first b = 0 image volume and 15 DWI volumes were used as the input data for fitting the tensor model.
- HCP‐2Shell: Two‐shell data consisting of all 18 b = 0 image volumes and 90 DWI volumes at both b = 1000 and 2000 s mm^−2^ were used for computing reference kurtosis parameters and DKI metrics. A subset of the first three b = 0 image volumes, 10 DWI volumes at b = 1000 s mm^−2^ and 20 DWI volumes at b = 2000 s mm^−2^ were used as the input data for fitting the kurtosis model.
- HCP‐3Shell: Three‐shell data consisting of all 288 image volumes were used for computing reference NODDI parameters and metrics. A subset of the first three b = 0 image volumes, 15 DWI volumes at b = 1000, 2000 and 3000 s mm^−2^ were used as the input data for fitting the NODDI model.

For each subject, the brain tissue mask generated from the T1w data using the FreeSufer software package (i.e., gray and white matter from the “aparc+aseg” segmentation) was resampled to the diffusion image space using nearest neighbor interpolation with an identity affine transformation.^[^ ^52^, ^53^ ^]^

#### 3.1.3 CDMD Diffusion Data

Pre‐processed diffusion data acquired at 2 mm isotropic resolution of one subject from the public MGH CDMD data were used to evaluate the generalization performance of DIMOND.

The data of the subject consisted of 50 b = 0 image volumes and 800 DWI volumes at eight b‐values for each of the two diffusion times. Only the first b = 0 image volume and 32 DWI volumes acquired at b = 1500 s mm^−2^ were used in this study. The acquisition protocols and pre‐processing pipelines have been described in detail previously.^[^ ^54^ ^]^ Similarly, the “aparc+aseg” segmentation result at T1w image space was used to generate the brain tissue mask at the diffusion image space.

### 3.2 DIMOND for DTI on Simulation Data

The efficacy of DIMOND was first evaluated using the simulation data. Specifically, DIMOND was used for fitting the tensor model non‐linearly on different numbers (i.e., seven to 55) of input signal intensities at the two SNR levels (i.e., 10 and 20). Since the simulation was only performed for each individual voxel, convolution layers were not used in DIMOND's network for incorporating spatial information (i.e., *M* = 0, *N* = 7 for the NN). Dropout layers were also excluded for demonstrating the benefits of MC dropout. Output channel number of the first and the last layer was set to *c_1_* = 32 and *c_4_* = 7.

DTI model fitting involved the estimation of seven parameters ***P *** = [*D*_11_ *D*_22_ *D*_33_ *D*_12_ *D*_13_ *D*_23_ *S*_0_] ^*T*^, where *D_jk_* (*j*, *k* = 1, 2, 3) represents the six unique elements of the diffusion tensor, a 3 × 3 symmetric matrix, and *S*_0_ is the non‐diffusion‐weighted signal. The diffusion‐weighted signal *S_i_* along diffusion‐encoding direction ***v***_***i***_ = [*v*_*i*1_ *v*_*i*2_ *v*_*i*3_]  (*i* = 1, 2, 3, …, *n* where *n* is the number of image volumes) measured at b‐value *b_i_* was represented as:

(1) $$S_{i}=tensor\left(P,v_{i},b_{i}\right)=S_{0}e^{-b_{i}\sum_{j\;=\;1}^{3}\sum_{k\;=\;1}^{3}v_{ij}v_{ik}D_{jk}}$$

where *D_jk_* = *D_kj_*  since the tensor matrix is symmetric.^[^ ^10^ ^]^ The mean squared errors (MSEs) between all estimated and acquired signals defined as:

(2) $$L=\frac{1}{n}\sum_{i\;=\;1}^{n}MSE_{S_{i}\in V}\left(S_{i},{\hat{S}}_{i}\right)$$

were used for optimizing the parameters of network *G*, where $\hat{P}=G\left(S\right)$ and ${\hat{S}}_{i}=\mathrm{tens}\mathrm{or}\left(\hat{P},v_{i},b_{i}\right)$. ***S*** consisted of all observations [*S*_1_ … *S_n_*] of each individual voxel. V represented the set of *L* voxels used for the optimization, which included all voxels for the simulation data while excluding voxels within the cerebrospinal fluid (CSF) to optimize the network for brain tissues (i.e., white matter and gray matter) for the in vivo data.

For comparison, tensor parameters were also estimated using the benchmark ordinary least squares (OLS) regression method implemented in the widely adopted “dtifit” function of FMRIB Software Library software (FSL).^[^ ^55^, ^56^, ^57^ ^]^ The ground‐truth tensors and DTI metrics were estimated from all noise‐free data using FSL's “dtifit” function.

### 3.3 DIMOND for DTI on Empirical Data

The efficacy of DIMOND for fitting the tensor model on empirical data was then demonstrated using the HCP‐1Shell data. The input of the NN consisted of one b = 0 image volume and 15 DWI volumes. NNs excluded dropout layers were also evaluated for demonstrating the benefits of MC dropout. Output channel numbers of the first and last layer were set to *c_1_* = 32 and *c_4_* = 7.

Three training strategies were compared:

- One NN was trained on the data of each individual subject (i.e., subject‐specific training);
- An NN was pre‐trained on the data of all 10 subjects and then fine‐tuned on the data of each individual subject;
- An NN was pre‐trained on the data of a single subject and then fine‐tuned on the data of each individual subject.

Reference tensors and DTI metrics were computed from all b = 0 and b = 1000 s mm^−2^ data of each HCP subject using OLS regression implemented in FSL's “dtifit” function. Reference DWI volumes were synthesized from reference tensors via the tensor model.

The cross‐dataset generalization capability of DIMOND was assessed by directly applying the NN trained on the HCP data to the CDMD data, which were acquired with different diffusion‐encoding directions and b‐value (1000 s mm^−2^ for HCP vs 1500 s mm^−2^ for CDMD), spatial resolution (1.25 mm iso. for HCP vs 2 mm iso. for CDMD), and hardware systems. Because the contrast and number of DWI volumes of the two datasets were different, synthesized DWI volumes along the 32 diffusion‐encoding directions used for acquiring the CDMD data were generated from the reference diffusion tensors derived from the data of a single HCP subject to train the NN using DIMOND. This trained NN was also fine‐tuned on the data of the CDMD subject. For comparison, an NN initialized with random parameters was also trained on the data of the CDMD subject.

### 3.4 DIMOND for DKI

The benefits of DIMOND for fitting the more complex diffusion kurtosis model were demonstrated using the HCP‐2Shell data. Specifically, DIMOND was used to fit kurtosis model to 33 input image volumes in a subject‐specific training manner. Output channel numbers of the first and last layer were set to *c_1_* = 64 and *c_4_* = 22.

The kurtosis model required to estimate 22 parameters ***P *** = [***D*** ^***T***^ ***K*** ^***T***^ *S*_0_]^***T***^****** including six for diffusion coefficient (***D***), 15 for diffusion kurtosis (***K***) and one for non‐diffusion‐weighted signal (*S*_0_). The forward model to represent diffusion signal *S_i_* along diffusion‐encoding direction ***v***_***i***_ = [*v*_*i*1_ *v*_*i*2_ *v*_*i*3_]  (*i* = 1, 2, 3, …, *n*) with b‐value *b_i_* was:

(3) $$S_{i}=kurtosis\left(P,v_{i},b_{i}\right)=S_{0}e^{-b_{i}\sum_{j\;=\;1}^{3}\sum_{k\;=\;1}^{3}v_{ij}v_{ik}D_{jk}+\frac{1}{6}{b_{i}}^{2}{\left(\frac{1}{3}\sum_{j\;=\;1}^{3}D_{jj}\right)}^{2}K_{i}^{app}}$$

(4) $$K_{i}^{app}=\sum_{j=1}^{3}\sum_{k=1}^{3}\sum_{l=1}^{3}\sum_{m=1}^{3}v_{ij}v_{ik}v_{il}v_{im}K_{jklm}$$

where $K_{i}^{app}$ determines the sign of the apparent excess kurtosis ($K_{i}^{APP}$) along direction ***v***_***i***_, *D_jk_* = *D_kj_*  and *K_jklm_* = *K*_*sort*(*j*,*k*,*l*,*m*)_ .^[^ ^10^ ^]^ The loss function for the optimization was MSE as described in Equation (2) with $\hat{P}=G\left(S\right)$, ${\hat{S}}_{i}=\mathrm{kurt}\mathrm{osis}\left(\hat{P},v_{i},b_{i}\right)$ and V in brain tissues excluding voxels within CSF.

Moreover, the efficacy of DIMOND using more sophisticated loss functions for training the network was demonstrated. Specifically, the constrained weighted least squares (CWLS) regression method adopted in the “dki_fit” function of the MATLAB‐based DESIGNER (Diffusion parameter EStImation with Gibbs and NoisE Removal) software^[^ ^58^ ^]^ (https://github.com/NYU‐DiffusionMRI/DESIGNER) was used for training DIMOND's NN for DKI. Specifically, WLS regression conducted a point‐wise optimization employing a weighted least squared loss function:^[^ ^43^, ^59^ ^]^

(5) $$L_{\mathrm{cont}\mathrm{ent}}=\omega^{2}||\ln S-\ln{\hat{S}}^{\mathrm{cwls}}|{|}_{2}^{2}$$

(6) $$\omega =\mathrm{diag}\left(\exp\left(X{\hat{P}}^{\mathrm{ols}}\right)\right)=\mathrm{diag}\left({\hat{S}}^{\mathrm{ols}}\right)$$

subject to the lower bound constraint^[^ ^43^ ^]^ which assumes the non‐negativity of the apparent excess kurtosis and is mathematically formulated as:

(7) $$K_{i}^{\mathrm{app}}>0,i=1,2,3,\text{…},60$$

The ${\hat{P}}^{\mathrm{ols}}$ in Equation (6) was calculated by:

(8) $${\hat{P}}^{\mathrm{ols}}={\left(X^{T}X\right)}^{-1}\;X^{T}\ln S$$

where **X** (1×22) in Equations (6) and (8) is the design matrix constructed according to Equation (3). The $K_{i}^{app}$ (*i* = 1, 2, 3, …, 60) in Equation (7) was calculated along 60 uniformly distributed diffusion‐encoding directions. The WLS loss described in Equation (5) and the non‐negative constraint described in Equation (7) have been demonstrated effective in removing noise and avoiding physiological outliers.^[^ ^43^, ^59^ ^]^

Equations (5) and (7) were slightly modified for optimizing NNs. For Equation (5), the weights **ω** of each voxel were normalized since the contribution of each voxel to the network optimization was assumed equal:

(9) $$L_{content}=\sum_{i=1}^{n}MSE_{S_{i}\in V}\left(\sqrt{\omega_{S_{i}}}S_{i},\sqrt{\omega_{S_{i}}}{\hat{S}}_{i}\right)$$

(10) $$\omega_{S_{i}}=\frac{\exp\left({\hat{S}}_{i}^{\mathrm{ols}}\right)}{\sum_{j=1}^{n}\exp\left({\hat{S}}_{j}^{\mathrm{ols}}\right)}=\mathrm{soft}\max{\left({\hat{S}}^{\mathrm{ols}}\right)}_{i}$$

The lower bound constraint described in Equation (7) was implemented as a regularization term in the loss function using the *ReLU* function that penalizes negative $K_{i}^{app}$:

(11) $$L_{bound}=\frac{1}{L}\sum_{K^{app}\in V}\sum_{i\;=\;1}^{60}ReLU\left(-K_{i}^{app}\right)$$

$ReLU(-K_{i}^{app})$ is a positive value if $K_{i}^{app}$ is negative, and zero otherwise.

Therefore, the CWLS loss function for optimizing the NN of DIMOND was:

(12) $$L_{cwls}=L_{content}+\lambda L_{bound}$$

where the hyperparameter λ controls the strength of regularization. λ was equal to 1000 in our experiments. λ can be empirically set to any values >1000. DIMOND using the loss function descried in Equation (12) was entitled DIMOND‐CW.

For comparison, the kurtosis model parameters were also estimated using the MRtrix3 software^[^ ^60^ ^]^ (https://www.mrtrix.org/) and the DESIGNER software^[^ ^58^ ^]^ in three different ways:

- MRtrix3‐OLS: using OLS regression as implemented in MRtrix3's “dwi2tensor” function with the “‐ols” option;
- MRtrix3‐IWLS: using iterative weighted least squares (IWLS) regression as implemented in MRtrix3's “dwi2tensor” function;
- DESIGNER‐CWLS: using CWLS regression as implemented in DESIGNER's “dki_fit” function.

The MRtrix3‐IWLS improves upon MRtrix3‐OLS by using the iteratively updated weight matrix to deal with noisy inputs. Specifically, the iteration number was set to 10 in the “‐iter” option in this study (entitled MRtrix3‐IWLS10).

Reference diffusion kurtoses and DKI metrics were estimated on all b = 0, b = 1000 s mm^−2^ and b = 2000 s mm^−2^ data of each HCP subject using MRtrix3‐OLS. Reference DWI volumes were synthesized from reference kurtoses via the kurtosis model.

### 3.5 DIMOND for NODDI

The benefits of DIMOND for fitting the microstructural model in NODDI were demonstrated using the HCP‐3Shell data. Specifically, DIMOND was employed to generate the NODDI model parameters from 48 input image volumes. Output channel numbers of the first and last layer were set to *c_1_* = 128 and *c_4_* = 5.

NODDI modeling required to estimate five parameters ***P *** = [*f_iso_* *f_ic_* ***µ*** ^***T***^ *κ*]^*T*^ , including one for isotropic volume fraction (*f_iso_*), one for intra‐cellular volume fraction (*f_ic_*), two for mean orientation (***µ***), and one for measuring orientation dispersion (concentration parameter *κ* in Watson distribution). Using ***P***, NODDI models three tissue compartments within each voxel, including intra‐cellular compartment (signal: *A_ic_* (***µ***,*κ*, ***v***_***i***_,*b_i_*), fraction: *f_ic_*), extra‐cellular compartment (signal: *A_ec_* (***µ***,*f_ic_*, ***v***_***i***_,*b_i_*), fraction: 1‐*f_ic_*) and cerebrospinal fluid (CSF) compartment (signal: *A_iso_*, fraction: *f_iso_*).^[^ ^14^ ^]^ The forward model to represent the diffusion signal *S_i_* along diffusion‐encoding direction ***v***_***i***_ = [*v*_*i*1_ *v*_*i*2_ *v*_*i*3_]  (*i* = 1, 2, 3, …, *n*) measured at b‐value *b_i_* with non‐diffusion‐weighted signal *S*_0_ was:

(13) $$\begin{array}{ccc} S_{i} & = & \mathrm{NODDI}\left(P,v_{i},b_{i},S_{0}\right)=S_{0}\left(\left(1-f_{\mathrm{iso}}\right)\left(f_{\mathrm{ic}}A_{\mathrm{ic}}+\left(1-f_{\mathrm{ic}}\right)A_{\mathrm{ec}}\right)\right.\\ & & \left.+\;f_{\mathrm{iso}}A_{\mathrm{iso}}\right) \end{array}$$

The non‐diffusion‐weighted signal *S*_0_ was computed by averaging all acquired b = 0 image volumes, following the convention of other implementations. The forward model of NODDI in DIMOND followed the Dmipy implementation. The MSE between all estimated signals and acquired signals was defined in Equation (2), with $\hat{P}=\;G\left(S\right)$, ${\hat{S}}_{i}=\mathrm{NODDI}\left(\hat{P},v_{i},b_{i},S_{0}\right)$ and V including all voxels within the brain mask.

The network and training of DIMOND were slightly modified to adapt to NODDI model. First, since $\hat{\kappa }$ is larger than zero by definition, the corresponding network output *x* was scaled as below:

(14) $$\hat{\kappa }=\left\{\begin{array}{c} sigmoid\left(x\right),\;x<0\\ 0.5+x,\;x\geq 0 \end{array}\right.$$

Similarly, since ${\hat{f}}_{iso}$ and ${\hat{f}}_{ic}$ range between [0, 1], the corresponding network outputs were scaled using the sigmoid function.

Second, in order to optimize network parameters using gradient descent, the derivative function of confluent hypergeometric function *H*(0.5,1.5,*κ*) in *A_ic_* (***µ***,*κ*, ***v***_***i***_,*b_i_*) was implemented as:

(15) $$\frac{dH\left(0.5,1.5,\kappa \right)}{d\kappa }=\frac{1}{3}H\left(1.5,2.5,\kappa \right)$$

To demonstrate DIMOND's accuracy and efficiency for estimating NODDI model parameters, DIMOND's predictions were generated in three ways:

- One NN was trained on the data of each HCP subject with randomly initialized parameters and then applied to generate results for this particular subject (i.e., subject‐specific training);
- An NN was trained for a single subject as in (a), which was then directly applied to the data of other subjects to generate predictions;
- An NN was trained for a single subject as in (a), which was then fine‐tuned on the data of each subject and applied to generate results.

For comparison, NODDI model parameters were also estimated using the NODDI MATLAB Toolbox (NODDI‐Toolbox) shared by developers of the NODDI model (http://mig.cs.ucl.ac.uk/index.php?n = Tutorial.NODDImatlab) and the Dmipy software (https://github.com/AthenaEPI/dmipy) following the NODDI tutorial (https://nbviewer.org/github/AthenaEPI/dmipy/blob/master/examples/example_noddi_watson.ipynb).^[^ ^27^ ^]^

NODDI‐Toolbox employed a point‐wise grid search and gradient descent for the NODDI parameter estimation. The Dmipy implementation used a Brute–Force approach and utilized just‐in‐time compiler for acceleration. The computation of NODDI‐Toolbox and Dmipy were parallelized using 32 processes for acceleration on an Intel(R) Xeon(R) Platinum 8368Q CPU. The runtime for each HCP subject was recorded. Reference NODDI model parameters were estimated on all b = 0 and b = 1000, 2000, and 3000 s mm^−2^ data of each HCP subject using NODDI‐Toolbox.

### 3.6 Network Training

NNs and their optimization as well as the forward diffusion models were implemented using the PyTorch platform (https://pytorch.org/) and an NVIDIA A800 GPU. For both training and inference, input image volumes of the empirical data were split into blocks of size 64 × 64 × 64 × channels with minimal overlap to minimize the required GPU memory. Specifically, four and 12 blocks were extracted for the CDMD and HCP data, respectively. Data augmentation was performed by flipping each image block along the anatomical left‐right direction to avoid overfitting, especially for subject‐specific training. Therefore, the number of image blocks for training per epoch was eight and 24 for CDMD and HCP data, respectively. To improve the generalization of the network, input image volume intensities were normalized by dividing by the median value of the averaged b = 0 image volume intensities within brain tissues.

The optimization was performed using an Adam optimizer.^[^ ^61^ ^]^ The learning rate was adaptive to facilitate network convergence. For subject specific training, the learning rate was set to 0.001 for the first 10 000 blocks (e.g., 416 epochs for HCP data) and then decayed to 0.0001 for the following 5000 to 7000 blocks. For the pre‐training and fine‐tuning strategy, a learning rate of 0.001 and 0.0001 was used for each stage, respectively. For Monte Carlo validation, 80% image blocks were randomly selected for training and the rest 20% blocks for validation at each epoch. Monte Carlo validation was not used for NODDI since it made NN susceptible to local minima. The NN parameters corresponding to the minimum validation loss were recorded. The training speed was ≈20 blocks/s for tensor model and kurtosis model and was ≈10 blocks/s for NODDI model.

### 3.7 Result Evaluation

#### 3.7.1 Image quality

The structural similarity index (SSIM) and mean absolute error (MAE) were used to quantify the similarity of synthesized images compared to reference images within brain tissues for DTI and DKI. Reference images were generated from reference diffusion model parameters. For the calculation, intensities of each image were first standardized (i.e., subtracting the mean value of image intensities within brain tissues from the corresponding raw acquired image and then dividing by their standard deviation value) and then transformed to the range between 0 and 1 by adding three and dividing by six. The “ssim” function of MATLAB software was used to calculate the SSIM.

#### 3.7.2 Tensor Component Quality

The MAE within brain tissues was used to quantify the similarity between the resultant and reference tensor components. The voxel‐wise standard deviation of 20 inferences from DIMOND was computed to measure the MC dropout uncertainty of DIMOND results.

#### 3.7.3 Model Metric Quality

The MAEs between resultant and reference DTI metrics including axial diffusivity (AD), mean diffusivity (MD), fractional anisotropy (FA) and primary eigenvector (V1) within the brain tissue mask, DKI metrics including FA, MD and AD from the tensor component, mean kurtosis (MK), axial kurtosis (AK) and radial kurtosis (RK) from the kurtosis component within the brain tissue mask, and NODDI metrics including *f_iso_*, *f_ic_* and orientation dispersion index (ODI) within the brain mask were used to quantify the quality of diffusion model metrics. The group means and standard deviations of MAEs across 10 subjects were calculated.

The error for DTI V1 was calculated as the angle between the resultant and reference orientation vector. DKI metric errors beyond the 97th percentile were omitted for the MAE calculation due to numerous outliers in MK and RK maps generated by MRtrix3‐OLS and MRtrix3‐IWLS10. The outliers resulted from overfitting, leading to large negative values of the quartic *K^app^* along 256 directions employed for MK and RK calculation. In NODDI, when *f_iso_* approaches one, *f_ic_* and ODI can be of arbitrary values.^[^ ^14^ ^]^ Therefore, voxels with *f_iso_* higher than 0.8 were excluded for calculating MAEs of *f_ic_* and ODI.

## 4 Results

**Figure 3:**
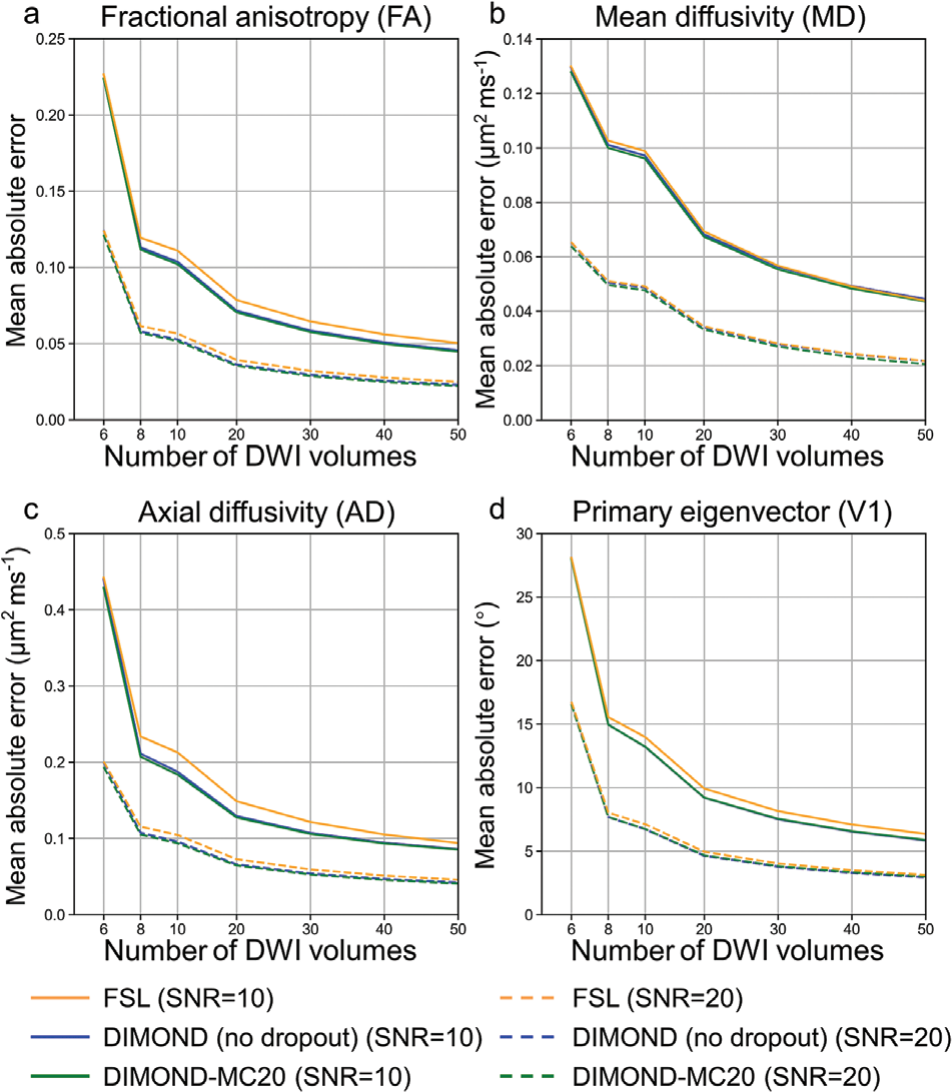
Simulation results. The mean absolute errors (MAEs) of a) DTI fractional anisotropy (FA), b) mean diffusivity (MD), c) axial diffusivity (AD), and d) principal eigenvector (V1) between the FSL results on noise‐free data (i.e., ground truth) and those from FSL (orange lines), DIMOND without dropout (blue lines), and DIMOND‐MC20 with MC dropout with a dropout rate of 0.05 (green lines) using different numbers of input diffusion‐weighted image (DWI) volumes (i.e., 6, 8, 10, 20, 30, 40 and 50) at SNR = 10 (solid lines) and SNR = 20 (dashed lines) are plotted.

DIMOND generated high‐quality DTI metrics on the simulation data. The MAEs compared to the ground truth for FA, AD, MD and V1 from DIMOND were consistently lower than those from FSL results (**Figure** **3** blue and green vs orange curves). The advantages of DIMOND over FSL were greater for noisier data (Figure 3 solid curves for SNR = 10 vs dashed curves for SNR = 20), indicating that DIMOND can effectively remove noise. The differences between DIMOND and FSL results for MD were subtle (Figure 3b), because MD is easier to estimate compared to other metrics. For FA, AD and V1 (Figure 3a,c,d), DIMOND results were more accurate than FSL results when the number of input DWI volumes ranged between 8 and 30, indicating that DIMOND's denoising effects were not obvious if the overall SNR of the input data was very low (e.g., six input DWI volumes) or was sufficient (e.g., >30 input DWI volumes). Finally, DIMOND results generated using MC dropout were slightly more accurate than those generated without dropout in the network (Figure 3 green vs blue curves).

**Figure 4:**
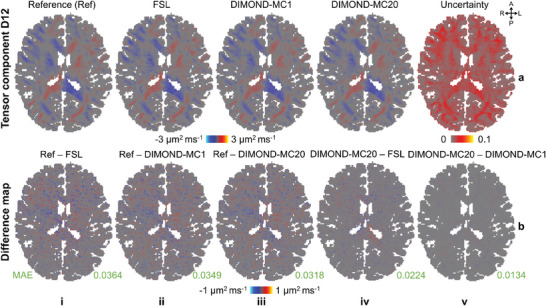
Tensor components. a) Exemplary axial image slices of the tensor component *D*_12_ generated from all available HCP‐1Shell data using FSL (reference, a(i)) and those generated from sub‐sampled data using FSL (a(ii)) and subject‐specific trained DIMOND (a(iii) and a(iv)), as well as the uncertainty map measured from the 20 inferences (a(v)) are shown. b) The difference maps between the reference and FSL result from sub‐sampled data (b(i)), reference and DIMOND result (b(ii) and b(iii)), DIMOND‐MC20 and FSL results (b(iv)), and DIMOND‐MC20 and DIMOND‐MC1 results (b(v)) are shown. The mean absolute errors (MAE, green) are listed to quantify similarity between tensor components.

Furthermore, DIMOND recovered high‐quality tensor component *D*_12_ from sub‐sampled empirical data. The tensor component maps generated from DIMOND‐MC20 and DIMOND‐MC1 were visually cleaner, more similar to the reference than that from FSL (**Figure** **4**a, (iv,iii) vs (ii) and Figure 4b, (iii,ii) vs (i), MAE: 0.0318 µm^2^ ms^−1^, 0.0349 µm^2^ ms^−1^ vs 0.0364 µm^2^ ms^−1^). The difference maps of DIMOND and FSL results compared to the reference did not contain anatomical structures and were similar (Figure 4b(i‐iii)). Averaging multiple inferences using MC dropout reduced the variance of predictions and improved DIMOND‐MC20 result upon DIMOND‐MC1 result (Figure 4b(ii,iii), MAE: 0.0349 to 0.0318 µm^2^ ms^−1^). The difference was subtle (Figure 4b(v), MAE: 0.0134 µm^2^ ms^−1^), indicating low uncertainty. Results of *D*_11_ are available in Figure S1 (Supporting Information).

**Figure 5:**
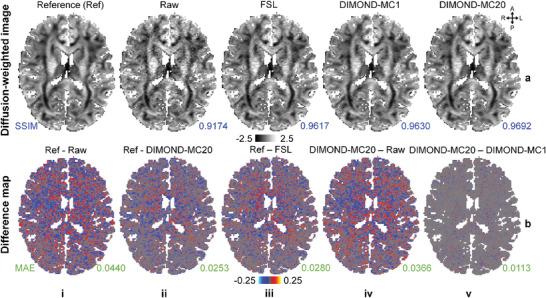
Images synthesized using tensor model. a) Exemplary axial image slices of diffusion‐weighted image (DWI) volumes (b = 1000 s mm^−2^ along [0.20, 0.89, −0.40]) from reference data (a(i), synthesized using reference tensors via the tensor model), raw acquired data (a(ii)), and those synthesized from tensors generated using FSL (a(iii)) and subject‐specific trained DIMOND (a(iv) and a(v)) from sub‐sampled data of a representative HCP subject are shown. b) Difference maps between the reference and raw acquired DWI (b(i)), reference and DIMOND‐MC20 result (b(ii)), reference and FSL result (b(iii)), DIMOND‐MC20 result and raw acquired DWI (b(iv)), and DIMOND‐MC20 and DIMOND‐MC1 results (b(v)) are shown. The structural similarity indices (SSIM, blue) are listed to quantify the similarity between displayed DWIs and the reference. The mean absolute errors (MAE, green) are listed to quantify the difference between DWIs.

DWIs synthesized from resultant tensors further demonstrated the denoising capability of DIMOND. DWIs from DIMOND‐MC20 and DIMOND‐MC1 were cleaner than the raw acquired DWI as well as the DWI from FSL and therefore were more similar to the reference (**Figure** **5**a, (v) vs (iv) vs (ii) vs (iii)). All difference maps did not contain anatomical structures (Figure 5b). The noise patterns represented in the difference maps between the reference and DIMOND‐MC20 result and those between the reference and FSL result were overall similar (Figure 5b, (ii) vs (iii)). Finally, the difference between DIMOND‐MC20 and DIMOND‐MC1 results indicated that averaging multiple inferences in MC dropout further removed noise slightly (Figure 5a, (v) vs (iv) and Figure 5b(v)). Results of non‐diffusion‐weighted images are available in Figure S2 (Supporting Information).

**Figure 6:**
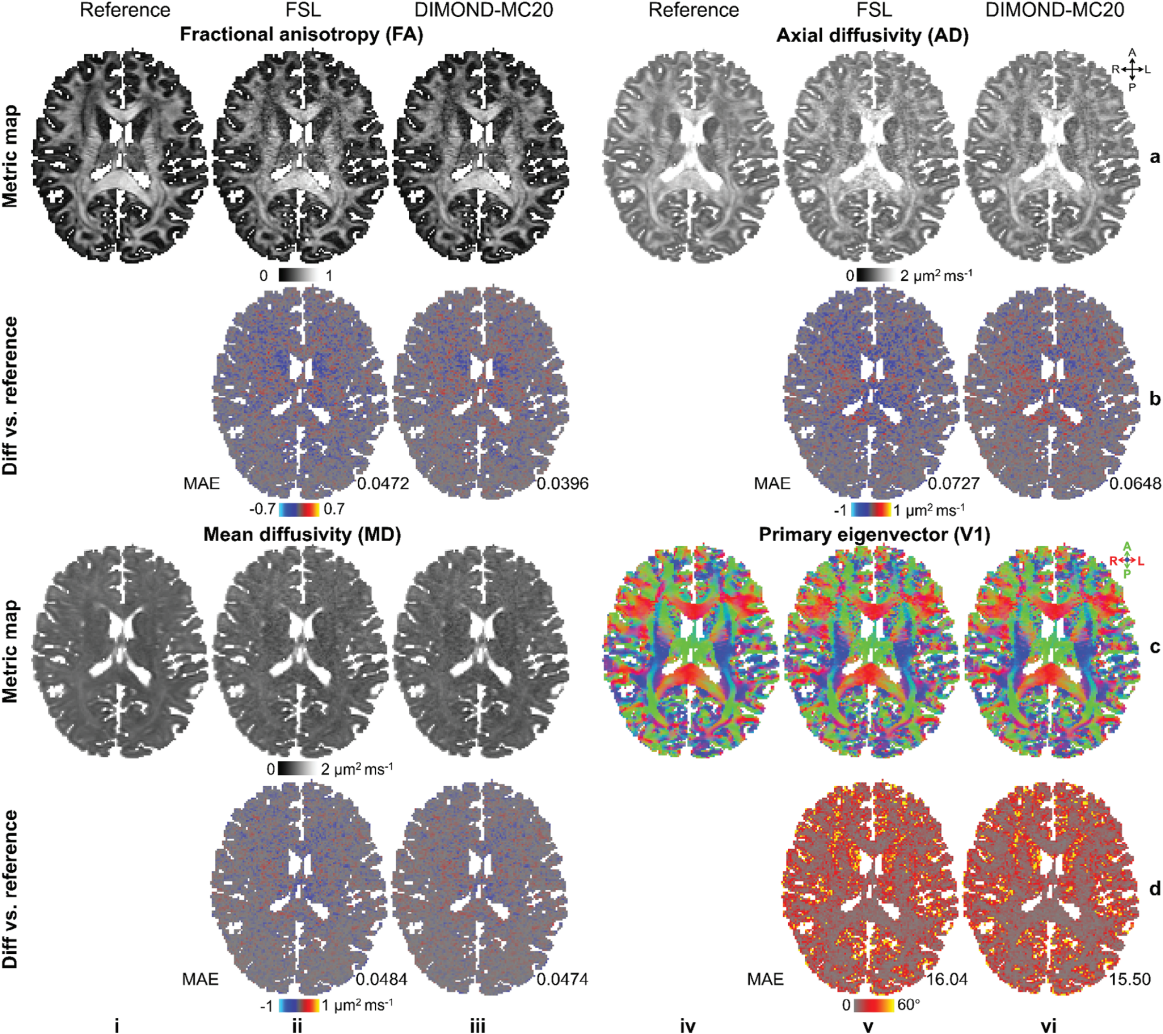
DTI metrics. a,c) Exemplary axial maps of fractional anisotropy (a(i‐iii)), axial diffusivity (a(iv–vi)), mean diffusivity (c(i–iii)) and primary eigenvector (c(iv‐vi)) derived from the reference tensors (i,iv), and tensor results generated from sub‐sampled data using FSL (ii,v) and subject‐specific trained DIMOND (iii,vi) of a representative HCP subject are shown. b,d) The difference maps compared to the reference are displayed, with the mean absolute error (MAE) of each residual map listed.

In addition to high‐quality tensor components and denoised images, DTI metrics from DIMOND were also accurate. The FA, AD and V1 maps from DIMOND‐MC20 were visually less noisy than those from FSL and more similar to the reference map (**Figure** **6**a(i–vi),c(iv–vi)). The difference for MD was subtle (Figure 6c(i–iii)). Quantitatively, the group means (± group standard deviations) of MAE from DIMOND‐MC20 were substantially lower than those from FSL in terms of all DTI metrics (**Table** **1**a–d, (iii) vs (i)), representing 17.3%, 9.6%, 0.6%, and 3.8% improvement for FA, AD, MD, and V1, respectively. Compared to DIMOND without dropout (Table 1ii), DIMOND‐MC20 improved the accuracy of FA and AD prediction (6.7% to 17.3% for FA, 7.5% to 9.6% for AD) but increased the MAE for MD and V1 slightly.

**Table 1:** DTI metric accuracy quantification. The group means (± group standard deviations) of mean absolute errors (MAE) of DTI metrics within the brain tissue between the reference and those from i) FSL, ii) DIMOND without dropout, and iii) DIMOND with MC dropout (DIMOND‐MC20 for 20 inferences and 0.05 dropout rate) across the ten healthy subjects of the HCP‐1 Shell data are listed. The red text and blue text highlight the lowest and second lowest MAEs.

		i	ii	iii
		FSL	DIMOND (no dropout)	DIMOND‐MC20 (dropout rate = 0.05)
a	FA	0.0539 ± 0.0043	0.0503 ± 0.0046	0.0446 ± 0.0036
b	AD (µm^2^ ms^−1^)	0.0763 ± 0.0045	0.0706 ± 0.0043	0.0690 ± 0.0039
c	MD (µm^2^ ms^−1^)	0.0511 ± 0.0032	0.0505 ± 0.0034	0.0508 ± 0.0033
d	V1 (°)	17.414 ± 0.96	16.738 ± 1.00	16.747 ± 0.87

**Figure 7:**
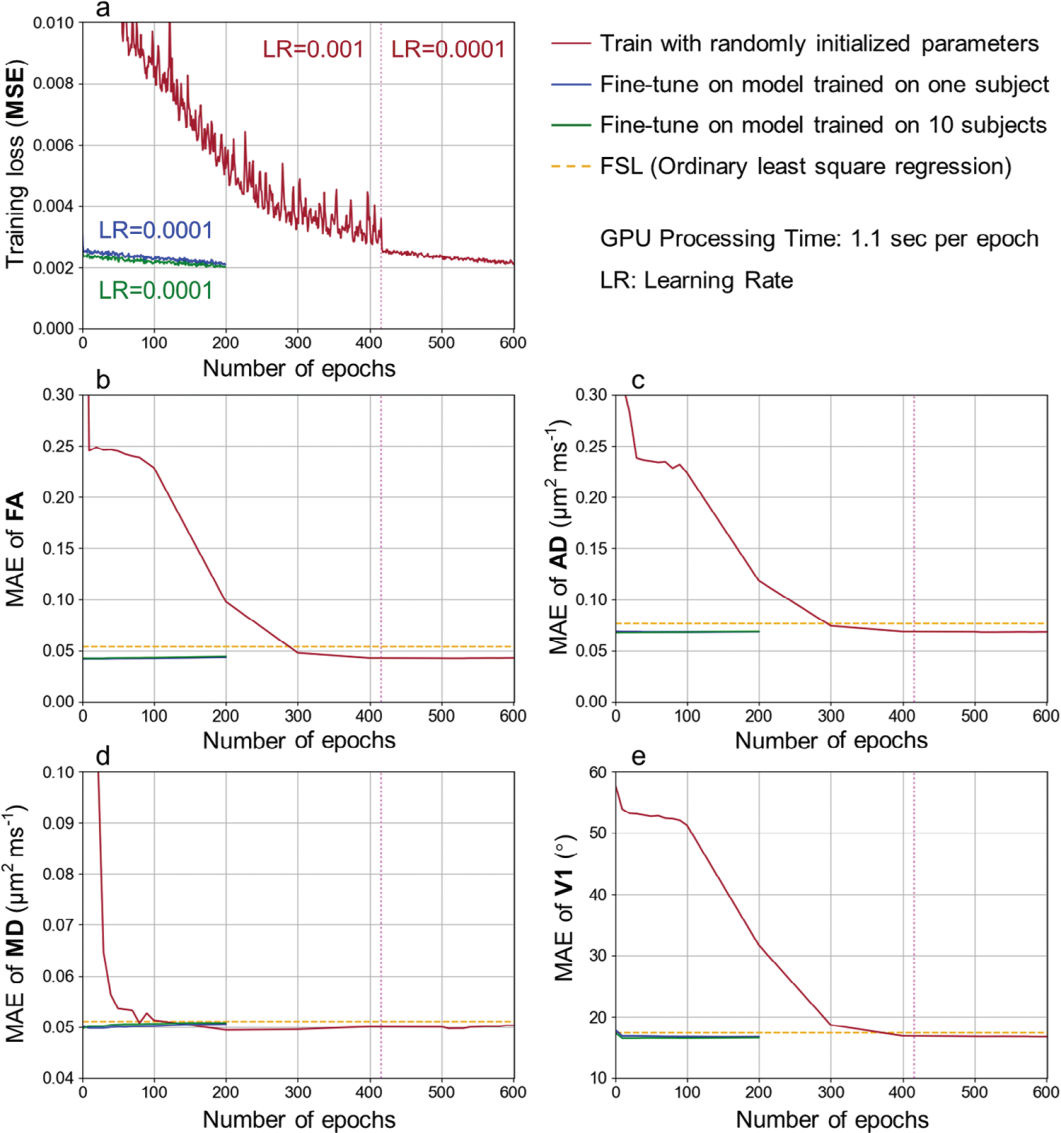
Cross‐subject generalization. The group means of a) the training loss and the mean absolute errors (MAEs) of b) DTI fractional anisotropy (FA), c) axial diffusivity (AD), d) mean diffusivity (MD) and e) principal eigenvector (V1) between reference metrics and those from sub‐sampled data using FSL (orange lines) and DIMOND‐MC20 of 10 HCP subjects over different numbers of training epochs are plotted. DIMOND‐MC20 network parameters were 1) randomly initialized and then optimized on the data of each subject (i.e., subject‐specific training, red lines, 0.001 learning rate between 0 and 416 epochs and 0.0001 afterward), 2) initialized with parameter values from the network pre‐trained on the data of a representative HCP subject and then fine‐tuned on the data of each subject (blue lines, 0.0001 learning rate), or 3) initialized with parameter values from the network pre‐trained on data of all 10 subjects and then fine‐tuned on the data of each subject (green lines, 0.0001 learning rate). The network parameters were recorded every 10 epochs (between 0 and 100 epochs) and then every 100 epochs (between 100 and 600 epochs) for predicting DTI metrics.

DIMOND exhibited great cross‐subject generalization. When directly applying NNs pre‐trained on data of all 10 subjects (**Figure** **7**, green lines) or a representative subject (Figure 7, blue lines), the training loss and MAEs for DTI metrics from DIMOND‐MC20 were similar to those from the converged subject‐specific training network (Figure 7, red lines) and lower than those from FSL (Figure 7, orange lines), except for MD. The high generalization capability rendered it feasible to fine‐tune pre‐trained NNs on the data of each individual subject to shorten the training time. After fine‐tuning, the training loss decreased while MAEs of DTI metrics stayed almost unchanged, with MAEs for MD and V1 slightly increased (Figure 7d) and decreased (Figure 7e), respectively.

**Figure 8:**
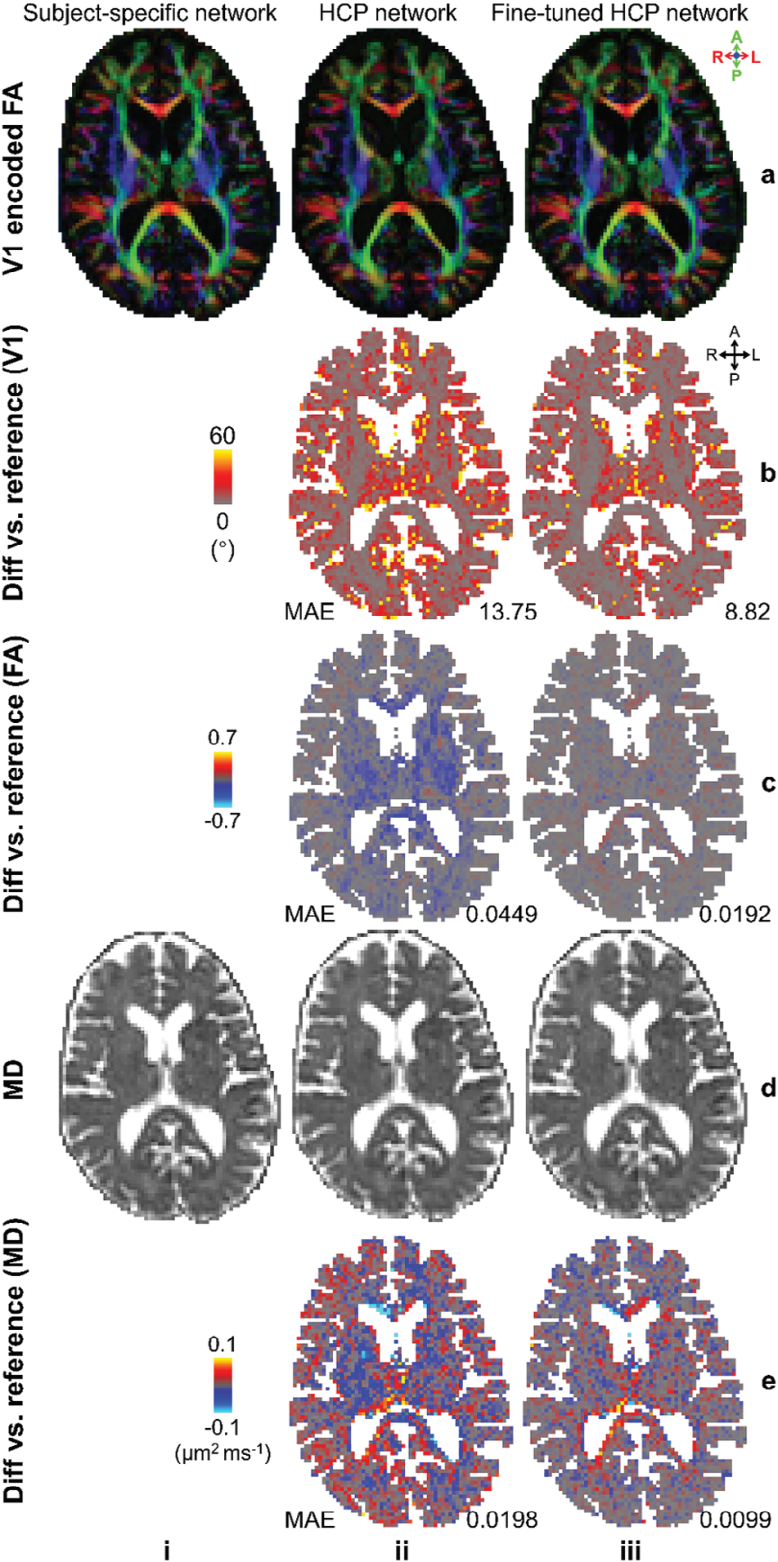
Cross‐dataset generalization. Exemplary axial image maps of a) primary eigenvector encoded fractional anisotropy (V1 encoded FA) and d) mean diffusivity (MD) generated by DIMOND‐MC20 using i) the subject‐specific trained neural network, ii) network trained on HCP data, and iii) network trained on HCP data and then fine‐tuned on the data of each individual subject of a representative CDMD subject are displayed. b,c,e) The difference maps compared to results from subject‐specific training are displayed, with the mean absolute errors (MAEs) listed to quantify the similarity.

DIMOND also generalized well across datasets acquired with distinct spatial resolutions, b‐values, and diffusion‐encoding directions. When directly applying networks trained on HCP data to CDMD data, resultant maps of V1 encoded FA and MD were visually highly similar to results from subject‐specific training (**Figure** **8** (i) vs (ii)). After fine‐tuning HCP networks on data of each individual CDMD subject, the difference compared to results from subject‐specific training was considerably reduced (Figure 8 (ii) vs (iii)).

**Figure 9:**
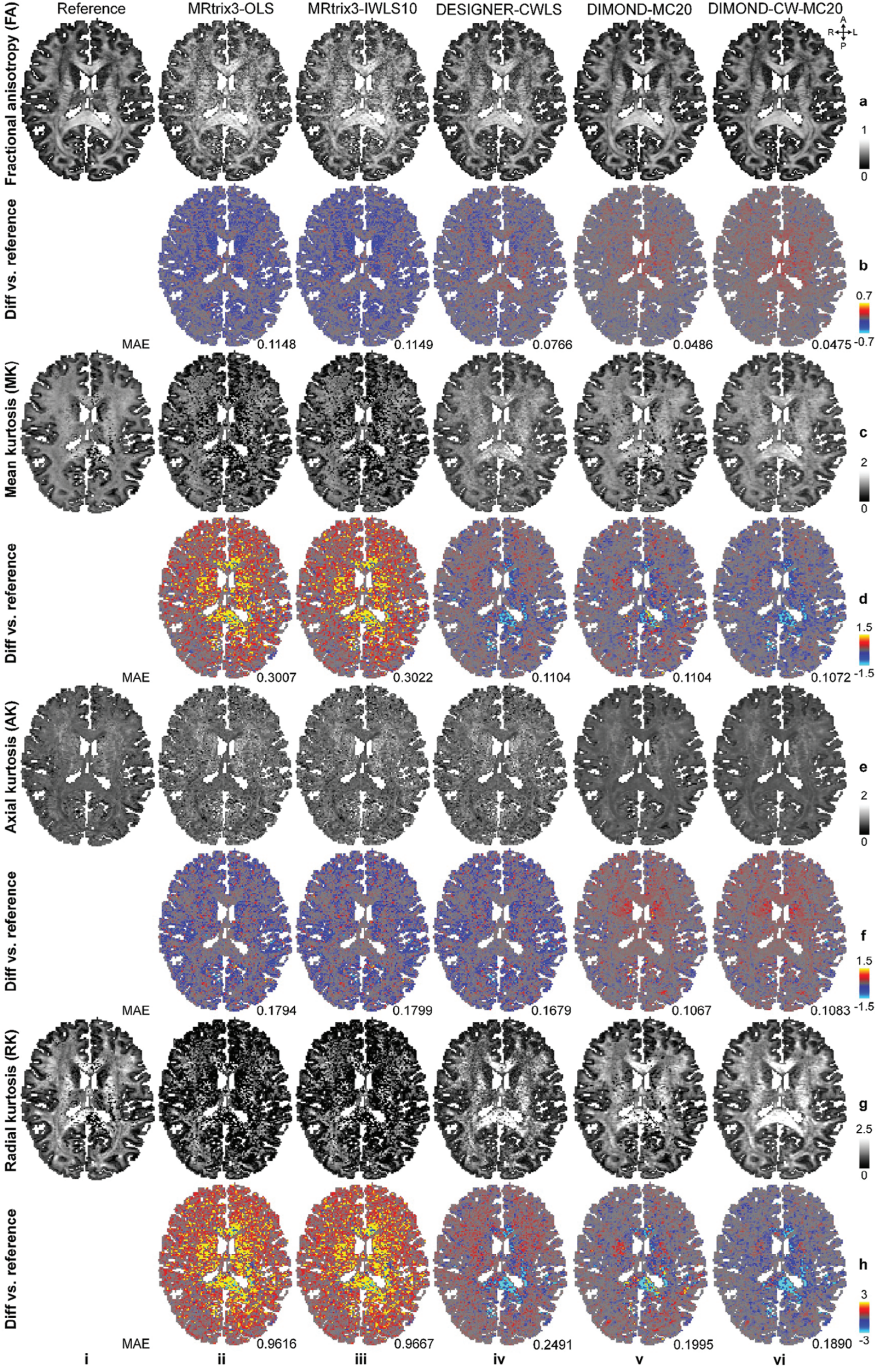
DKI metrics. Exemplary axial maps of DKI metrics including a) fractional anisotropy, c) mean kurtosis, e) axial kurtosis, and g) radial kurtosis derived from kurtosis results generated from i) MRtrix3‐OLS using all available HCP‐2Shell data (reference), and those generated from ii) MRtrix3‐OLS, iii) MRtrix3‐IWLS10, iv) DESIGNER‐CWLS, v) DIMOND‐MC20, and vi) DIMOND‐CW‐MC20 using sub‐sampled data of a representative HCP subject are shown. b,d,f,h) The difference maps compared to the reference are also displayed, with mean absolute errors (MAEs) listed to quantify the similarity.

In addition to the tensor model, DIMOND successfully fitted the kurtosis model and outperformed conventional methods. The FA map of DIMOND‐CW‐MC20 (**Figure** **9**a,b(vi), MAE: 0.0475) was most similar to the reference with lowest MAE and structural details preserved, followed by DIMOND‐MC20 (Figure 9a,b(v), MAE: 0.0486). Resultant FA maps from conventional methods were noisy (Figure 9a,b(ii‐iv), MAE: 0.1148, 0.1149, 0.0766). MRtrix3‐OLS and MRtrix3‐IWLS10 failed to estimate MK and RK values for numerous brain voxels not only on the 6‐fold accelerated data (Figure 9c,g(ii,iii), black pixels), but also on all available HCP‐2Shell data (Figure 9c,g(i)). DESIGNER‐CWLS and DIMOND‐CW‐MC20 generated MK, AK, and RK maps with much fewer outliers due to the lower bound constraint (Equation (11)). Results from DIMOND‐CW‐MC20 were less noisy with remarkably lower MAE. Even though DIMOND‐MC20 only used the simple MSE loss for the optimization, its performance was competitive and outperformed DESIGNER‐CWLS (Figure 9 (v) vs (iv)). DIMOND‐CW‐MC20 slightly improved upon DIMOND‐MC20 (Figure 9 (vi) vs (v)).

Quantitatively, the group means of MAEs for DKI metrics derived from DIMOND‐CW‐MC20 were substantially lower than those from other methods (**Table** **2** (v) vs (i–iv) and **Table** **3**). The relative reductions of MAE for RK were more than 90% compared to those from MRtrix3‐OLS and MRtrix3‐IWLS10 (Table 3f(i,ii)). The MAEs of DIMOND‐MC20 achieved the second lowest MAEs in FA, MD, AD, AK and RK (Tables 2iv and 3iv). DESIGNER‐CWLS slightly outperformed DIMOND‐MC20 in terms of MK, presumably due to the use of lower bound constraint (Table 2d, (iii) vs (iv) and Table 3d, (iii) vs (iv)).

**Table 2:** DKI metric accuracy quantification. The group means (± group standard deviations) of the mean absolute error (MAE) of DKI metrics between the reference and those from i) MRtrix3‐OLS, ii) MRtrix3‐IWLS10, iii) DESIGNER‐CWLS, iv) DIMOND‐MC20, and v) DIMOND‐CW‐MC20 across 10 HCP subjects are listed. The red text and blue text highlight the lowest and second lowest MAEs respectively.

	Method	i	ii	iii	iv	v
		MRtrix3‐OLS	MRtrix3‐IWLS10	DESIGNER‐CWLS	DIMOND‐MC20	DIMOND‐CW‐MC20
a	AD (µm^2^ ms^−1^)	0.154 ± 0.0100	0.154 ± 0.0100	0.139 ± 0.0083	0.0878 ± 0.0049	0.0873 ± 0.0051
b	MD (µm^2^ ms^−1^)	0.0649 ± 0.0038	0.0649 ± 0.0038	0.0607 ± 0.0034	0.0558 ± 0.0039	0.0546 ± 0.0038
c	FA	0.119 ± 0.0087	0.118 ± 0.0087	0.0765 ± 0.0038	0.0471 ± 0.0029	0.0467 ± 0.0024
d	MK	0.616 ± 0.310	0.608 ± 0.300	0.125 ± 0.014	0.128 ± 0.015	0.123 ± 0.015
e	AK	0.192 ± 0.0074	0.192 ± 0.0074	0.180 ± 0.0066	0.115 ± 0.0060	0.115 ± 0.0058
f	RK	2.993 ± 2.38	2.930 ± 2.29	0.285 ± 0.027	0.240 ± 0.034	0.228 ± 0.032

**Table 3:** Relative improvement. Relative reductions of the group‐mean mean absolute errors (MAEs) for different DKI metrics of DIMOND‐CW‐MC20 listed in Table 2 over those from i) MRtrix3‐OLS, ii) MRtrix3‐IWLS10, iii) DESIGNER‐CWLS, and iv) DIMOND‐MC20 are listed.

	Method	i	ii	iii	iv
		MRtrix3‐OLS	MRtrix3‐IWLS10	DESIGNER‐CWLS	DIMOND‐MC20
a	AD	43.3%	43.3%	37.2%	0.569%
b	MD	15.9%	15.9%	10.1%	2.15%
c	FA	60.8%	60.4%	39.0%	0.849%
d	MK	80.0%	79.8%	1.60%	3.91%
e	AK	40.1%	40.1%	36.1%	0.00%
f	RK	92.4%	92.2%	20.0%	5.00%

**Figure 10:**
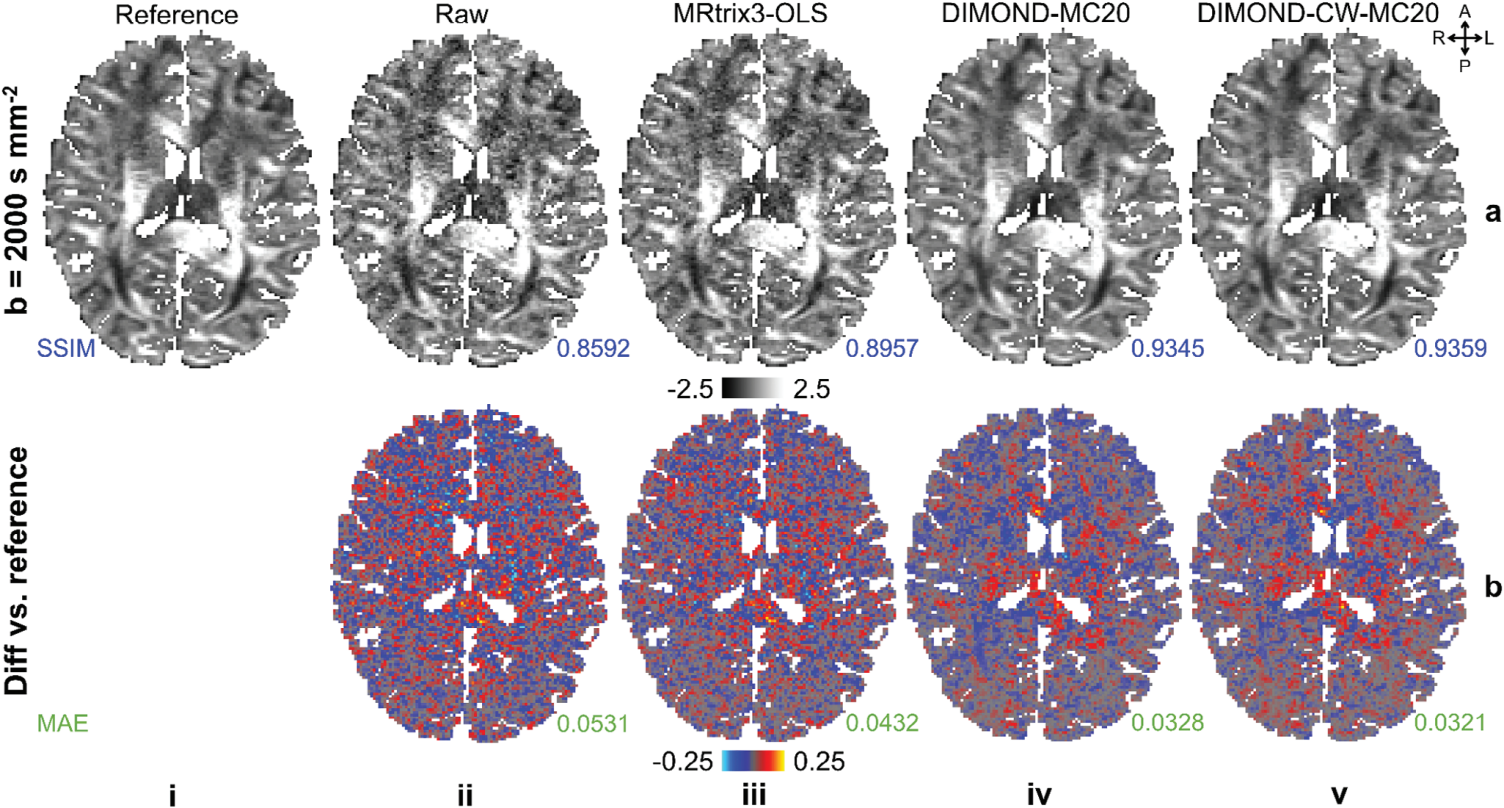
Images synthesized using kurtosis model. a) Exemplary axial image slices of diffusion‐weighted image (DWI) volumes (b = 2000 s mm^−2^ along [0.33, 0.87, 0.35]) from the reference data (a(i), synthesized using reference kurtoses via the kurtosis model), raw acquired data (a(ii)), and those synthesized from kurtoses generated using MRtrix3‐OLS (a(iii)), DIMOND‐MC20 (a(iv)), and DIMOND‐CW‐MC20 (a(v)) from sub‐sampled data of a representative HCP subject are shown. b) Difference maps between the reference and resultant DWIs are displayed, with structural similarity indices (SSIM, blue) and mean absolute errors (MAE, green) listed to quantify the difference.

High‐b‐value DWIs synthesized from DIMOND‐fit kurtosis model were also more accurate and cleaner. DWIs of b = 2000 s mm^−2^ derived from DIMOND‐MC20 and DIMOND‐CW‐MC20 demonstrated similar image sharpness and substantially higher SNR compared to those from raw data and MRtrix3‐OLS (**Figure** **10**a, (v,iv) vs (iii)), similar to results for the tensor model (Figure 5). All difference maps compared to the reference did not contain any structural biases (Figure 10b). The image quality improvement of DIMOND‐CW‐MC20 upon DIMOND‐MC20 suggested the benefits of the constrained weighted loss function, as expected (Figure 10 (v) vs (iv)). Synthesized DWIs of b = 1000 s mm^−2^ are available in Figure S3 (Supporting Information).

**Figure 11:**
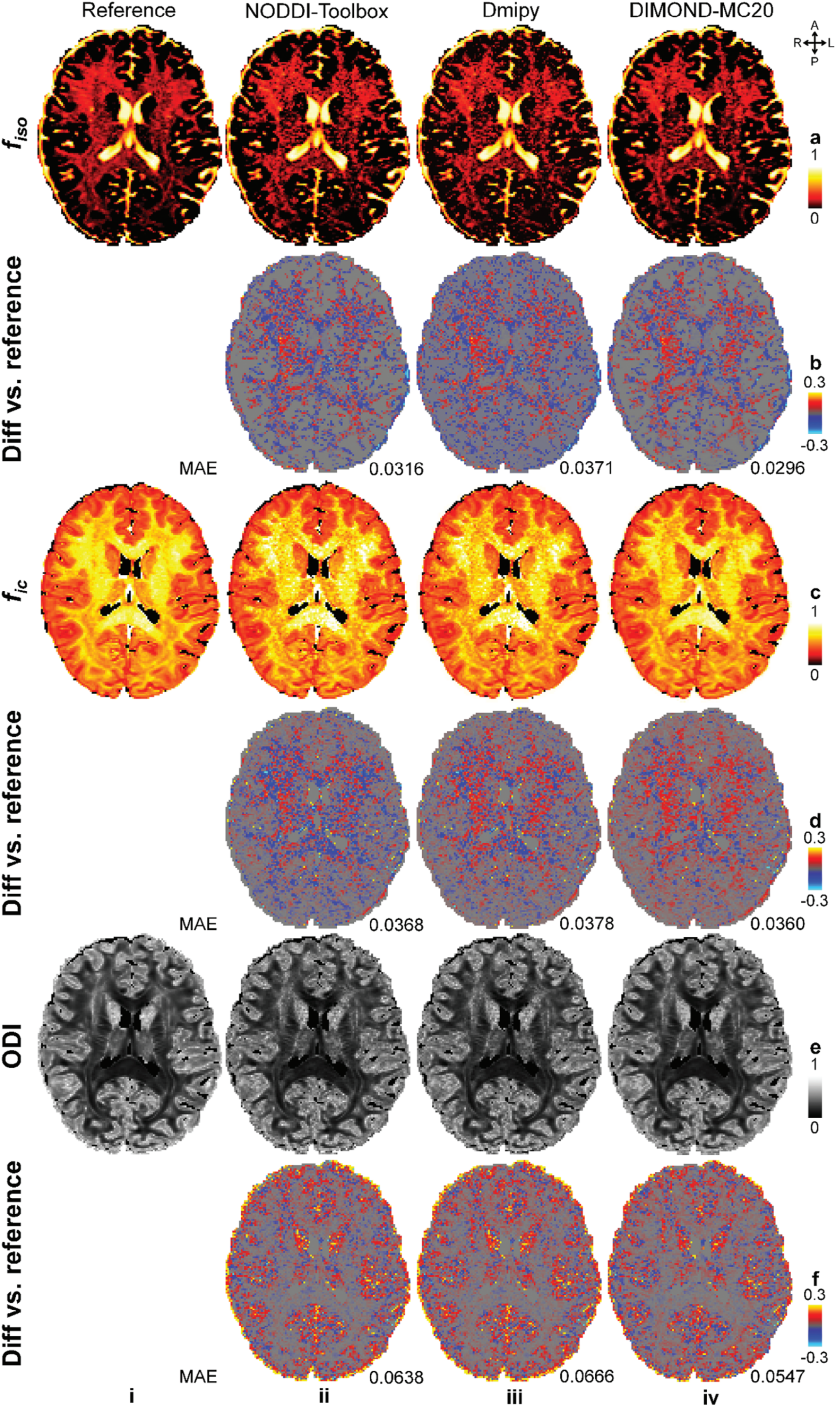
NODDI metrics. Exemplary axial maps of a) isotropic volume fraction (*f_iso_*), c) intracellular volume fraction (*f_ic_*), and e) orientation dispersion index (ODI) generated from i) NODDI‐Toolbox on all available HCP‐3Shell data (reference), and those generated using ii) NODDI‐Toolbox, iii) Dmipy, and iv) DIMOND‐MC20 from the sub‐sampled data of a representative HCP subject are shown. b,d,f) The residual maps compared to the reference are also displayed, with the mean absolute errors (MAE) listed to quantify the similarity.

In addition to diffusion signal representation, DIMOND fitted the sophisticated microstructural model in NODDI effectively and efficiently. DIMOND‐MC20 outperformed NODDI‐Toolbox and Dmipy in terms of all NODDI metrics (**Figure** **11**, (iv) vs (ii) and (iii)). The *f_iso_* map of DIMOND‐MC20 was most similar to the reference and free of noise. NODDI‐Toolbox tended to over‐estimate *f_ic_* values while Dmipy and DIMOND‐MC20 tended to under‐estimate *f_ic_* values (Figure 11d).

Quantitatively, the group means (± group standard deviations) of MAEs from DIMOND‐MC20 were substantially lower than those from NODDI‐Toolbox and Dmipy (**Table** **4**, (iii–v) vs (i,ii)). For instance, the group‐mean MAEs of the subject‐specific trained DIMOND‐MC20 (Table 4iii) decreased by 4.7%, 1.5%, 11.6% and 17.3%, 4.2%, 15.5% compared to NODDI‐Toolbox and Dmipy (Table 4i,ii), respectively, in terms of *f_iso_*, *f_ic_* and ODI. DIMOND‐MC20 results using subject‐specific training (Table 4iii), a network pre‐trained on the data of a representative HCP subject (Table 4iv), or a pre‐trained network further fine‐tuned on the data of each individual subject (Table 4v) were in general very similar. In terms of efficiency, DIMOND‐MC20 by directly applying a pre‐trained network only took 32 s for 20 inferences (i.e., 1.6 s per inference), remarkably faster than other methods especially NODDI‐Toolbox (i.e., 12.3 h).

**Table 4:** NODDI metric accuracy quantification. a‐c) The group means (± group standard deviations) of the mean absolute error (MAE) of NODDI metrics between the reference and those generated from i) NODDI‐Toolbox (parallel computing using 32 processes), ii) Dmipy (parallel computing using 32 processes) and iii‐v) DIMOND‐MC20 (network training and application on an A800 GPU) on the sub‐sampled HCP‐3Shell data across 10 HCP subjects are listed. For DIMOND, results were generated using a subject‐specific trained network (iii), a network pre‐trained on the data of a representative HCP subject (iv), or a pre‐trained network fine‐tuned on the data of each individual subject (v), 200 epochs for the fine‐tuning process). d) The group means (± group standard deviations) of time cost of each method are also listed. For DIMOND, the time cost included both the training time and inference time (iii,v) or only the inference time when training was not needed (iv). The red and blue text highlight the lowest and second lowest MAEs, or shortest and second shortest runtime.

		i	ii	iii	iv	v
		NODDI‐Toolbox (32 processes)	Dmipy implementation (32 processes)	DIMOND‐MC20 (subject‐specific trained network)	DIMOND‐MC20 (pre‐trained network)	DIMOND‐MC20 (fine‐tuned network)
a	*f_iso_* MAE	0.0321 ± 0.0018	0.0370 ±0.0016	0.0306 ± 0.0017	0.0303 ± 0.0018	0.0307 ± 0.0018
b	*f_ic_* MAE	0.0395 ± 0.0024	0.0406 ± 0.0026	0.0389 ± 0.0025	0.0393 ± 0.0026	0.0387 ± 0.0023
c	ODI MAE	0.0671 ± 0.0041	0.0702 ± 0.0043	0.0593 ± 0.0040	0.0583 ± 0.0033	0.0590 ± 0.0041
d	Runtime	12.3 ± 1.7 h	2.3 ± 0.4 h	24.1 ± 0.6 min	32 ± 3 s	7.9 ± 0.2 min

## 5 Discussion

This study proposes a new framework “DIMOND” for estimating diffusion model parameters using physics‐informed and self‐supervised deep learning. DIMOND harnesses NNs to map noisy diffusion data to diffusion model parameters and optimizes network parameters by minimizing the difference between acquired input data and those synthesized from the network outputs via the forward model. The efficacy of DIMOND was first demonstrated using the diffusion tensor model on simulation data and empirical HCP‐1Shell data. DIMOND generated more accurate and sharper DTI metric maps compared to the OLS regression algorithm implemented in FSL, especially in the presence of image noise (Figures 3 and 6, and Table 1). DIMOND also exhibited good generalization capabilities, both across HCP subjects and across HCP and CDMD datasets acquired with different hardware systems and imaging protocols, and therefore benefitted from transfer learning for reducing the training time (Figures 7 and 8). In addition to the tensor model, DIMOND was shown to fit more sophisticated models for DKI and NODDI efficiently and accurately. DIMOND outperformed conventional methods MRtrix3‐OLS, MRtrix3‐IWLS, and DESIGNER‐CWLS, especially coupled with CWLS loss for the DKI model optimization (Figure 9, and Tables 2 and 3). For NODDI, DIMOND not only generated more accurate metrics than those from conventional methods NODDI‐toolbox and Dmipy (Figure 11 and Table 4) but also reduced the fitting time from hours to minutes or even seconds (Table 4).

DIMOND substantially expedites diffusion model fitting, particularly for multi‐compartment microstructural models. For instance, DIMOND reduced the NODDI model fitting time for a high‐resolution HCP diffusion dataset from 12 h required by NODDI‐Toolbox and two hours required by Dmipy to 24 min, achieving 30‐ and 5‐fold acceleration, respectively. This efficiency gain is further augmented by transfer learning, since DIMOND's network exhibits decent generalizability (Figure 7 and Table 4). Directly applying a pre‐trained network further reduced the DIMOND runtime for NODDI to 32 s while maintaining high accuracy (Table 4). It is worth noting that DIMOND generated 20 inferences in this study to perform MC dropout. It can be 20 times faster for generating one inference, with marginally compromised performance compared to the average of 20 inferences. In other words, it only takes DIMOND 1.6 s to apply a pre‐trained network for producing high‐quality NODDI results that are more accurate than those from NODDI‐Toolbox and Dmipy, which achieves 27675‐ and 5175‐fold acceleration, respectively. Consequently, DIMOND renders it possible to generate and display microstructural maps from NODDI or other advanced models on the console computers during the scanning. If DIMOND is used for large‐scale neuroimaging studies such as the UK Biobank Imaging Study^[^ ^20^, ^21^ ^]^ and widely adopted in scientific and clinical applications, it has a high potential to save tremendous amount of computation and energy. In practice, it is recommended to first perform subject‐specific training on data of a representative subject or study‐specific training on data of several subjects for constructing an NN tailored for a particular diffusion imaging protocol and a set of hardware systems, and then fine‐tuning the pre‐trained NN on the data of incoming subjects. The fine‐tuning process takes several minutes but accounts for potential signal variations.

**Figure 12:**
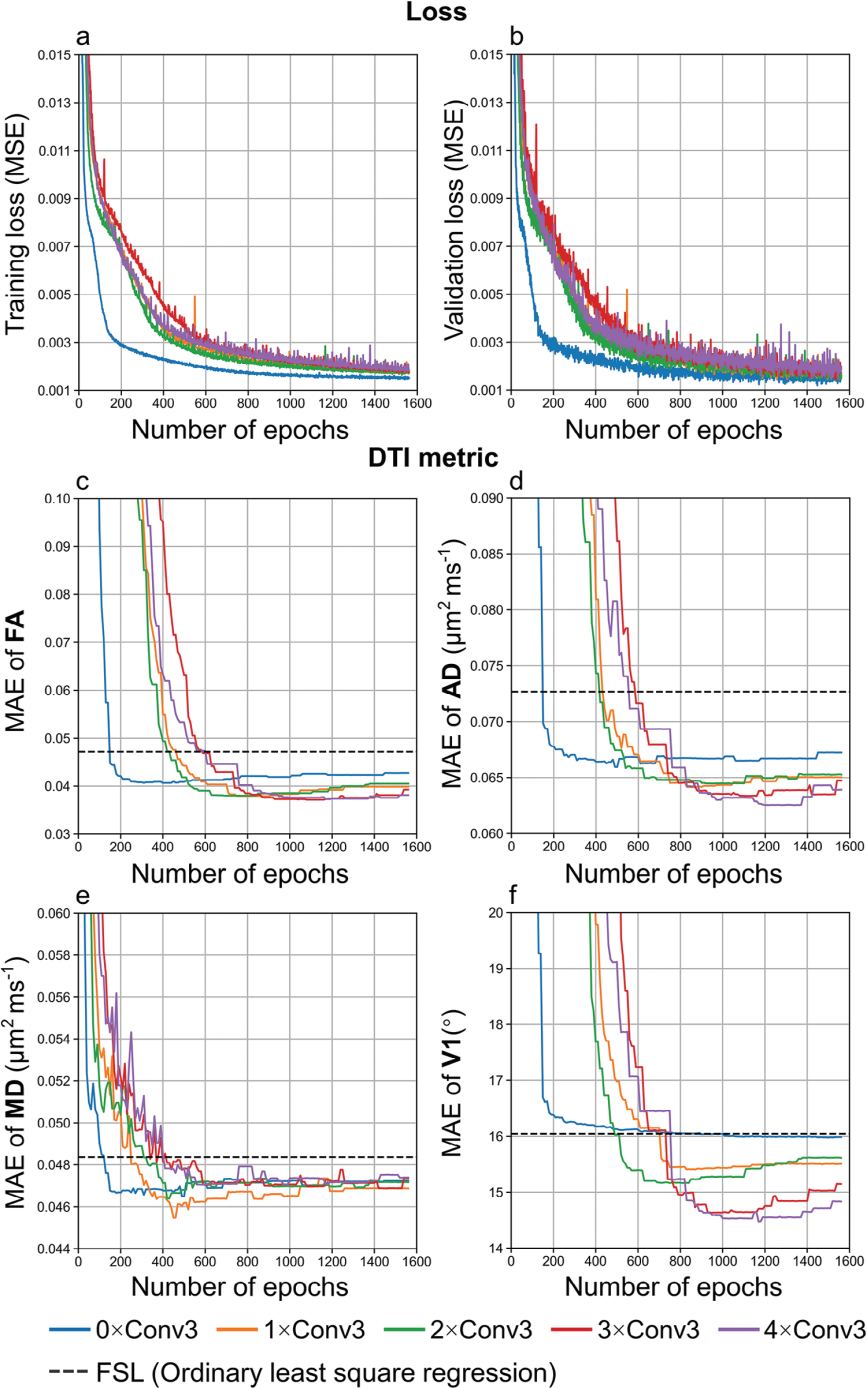
Effects of spatial information utilization. a) The training loss, b) validation loss, and c–f) mean absolute errors (MAEs) of DTI metrics between the reference and those from sub‐sampled data using FSL (dashed lines) and DIMOND‐MC20 using neural networks composed of zero to four 3 × 3 × 3 convolution layers of a representative HCP subject from HCP‐1Shell data over different number of training epochs are plotted. Network parameters were recorded every 10 epochs for predicting DTI metrics.

The efficiency and superior performance of DIMOND stem from the utilization of CNNs. As a universal function approximator,^[^ ^62^ ^]^ NNs effectively capture the intricate non‐linear relationship between noisy diffusion signals and model parameters and accurately approximate the mapping using a series of simple operations that can be efficiently executed on modern hardware systems, e.g., GPUs. Traditionally, model parameters are independently estimated for each image voxel, neglecting a variety of redundant information. In contrast, DIMOND's CNN learns to leverage similarity across local and non‐local voxels, diffusion‐encoding directions and b‐values, and potentially subjects, which greatly benefits to the reduction of noise in model estimates (Figures 4, 5, and 10) and required data for the model fitting. For empirical data, one 3 × 3 × 3 convolution layer was used to aggregate information from neighboring 27 voxels which exhibit highly similar microstructure and noise patterns. The spatial correlation significantly improved the accuracy of DTI metrics compared to those from network without convolution layers by 3.5% on average (**Table** **5** (iii) vs (ii) and **Figure** **12** orange lines vs blue lines). Using more convolution layers and nearby voxels for the model fitting only slightly improves model estimation accuracy by at most 1.6% on average (Table 5, (iii) vs (iv–vi)). One convolution layer leverages information from 27 (3 × 3 × 3) neighboring voxels, achieving 27‐fold increase compared to NNs without using convolution layer. The relative increase of the number of usable voxels by adding more convolution layers gradually slows down, e.g., about five‐fold from one to two convolution layers (i.e., 3 × 3 × 3 to 5 × 5 × 5 voxels) and 2.7‐fold from two to three convolution layers (i.e., 5 × 5 × 5 to 7 × 7 × 7 voxels). Considering that employing more convolution layers increases network parameters and computations, increasing NN's susceptibility to overfitting and slowing down training convergence (Figure 12), one convolution layer was used in this study and is recommended. Dropout layers were also adopted to avoid overfitting for subject‐specific training.

**Table 5:** DTI metric accuracy quantification. a,c,e,g) Lowest mean absolute errors (MAE) of curves in Figure 12 for different DTI metrics from DIMOND‐MC20 using neural networks composed of zero to four 3 × 3 × 3 convolution layers of a representative HCP subject from HCP‐1 Shell data are listed. b,d,f,h) The relative reduction in MAEs compared to FSL results are also reported. The red and blue text highlight the lowest and second lowest MAEs.

		i	ii	iii	iv	v	vi
		FSL	0×Conv3	1×Conv3	2×Conv3	3×Conv3	4×Conv3
a	FA	0.0472	0.0407	0.0379	0.0380	0.0371	0.0373
b		NA	13.77%	19.70%	19.49%	21.40%	20.97%
c	AD (µm^2^ ms^−1^)	0.0727	0.0659	0.0641	0.0645	0.0634	0.0625
d		NA	9.35%	11.83%	11.28%	12.79%	14.03%
e	MD (µm^2^ ms^−1^)	0.0484	0.0465	0.0455	0.0464	0.0469	0.0470
f		NA	3.93%	5.99%	4.13%	3.10%	2.89%
g	V1 (°)	16.043	15.978	15.404	15.162	14.632	14.473
h		NA	0.41%	3.98%	5.49%	8.80%	9.79%

Intuitively, DIMOND works by solving diffusion model fitting, a non‐linear optimization problem, using the deep learning framework. This concept has been demonstrated effective in image reconstruction,^[^ ^63^, ^64^ ^]^ image co‐registration,^[^ ^65^ ^]^ relaxometry,^[^ ^66^, ^67^ ^]^ and IVIM parameter estimation.^[^ ^68^, ^69^ ^]^ In contrast, this study comprehensively solves any diffusion MRI signal representation or modeling from multi‐b‐value and multi‐directional diffusion data. In addition to the efficiency and performance, the self‐supervised and physics‐informed deep learning‐based framework provides other benefits and represents the next‐generation diffusion model fitting methodology. First, DIMOND only requires the data of the subject or several subjects of a study for training the network, which reduces the requirement for collecting and processing additional training data and the risk for generalizing networks, especially for patients’ data with pathological image textures. The self‐supervised nature of DIMOND renders it user friendly to clinicians and neuroscientists lacking expertise in machine learning. Second, the development, deployment, and distribution of diffusion models using DIMOND become simpler. DIMOND can be implemented using general and open‐source machine learning frameworks such as PyTorch^[^ ^70^ ^]^ and Tensorflow,^[^ ^71^ ^]^ which provide numerous optimizers and loss functions as well as mature interfaces to hardware systems such as GPUs and those on the cloud. It is likely that developers and users only need to specify the forward model. The source codes of DIMOND will be made publicly available (https://github.com/Lthinker/DIMOND) and shared through open‐source software libraries such as Diffusion Imaging in Python (DIPY).^[^ ^48^, ^72^ ^]^ Finally, DIMOND, as a universal solver, also provides a way to evaluate and compare different diffusion models in terms of their capability of predicting input diffusion signals, i.e., the similarity between the raw acquired signals and the synthetic signals generated informed by the forward model.

There are still several limitations of this study. First, a plain NN is currently used which does not explicitly encode b‐values and gradient directions. Future work will focus on constructing layers that are aware of the diffusion‐encoding gradient information by using techniques such as attention calculation.^[^ ^73^ ^]^ Advanced NNs and deep learning technologies such as transformers^[^ ^74^ ^]^ and diffusion probabilistic models^[^ ^75^ ^]^ can be simply incorporated in the DIMOND framework to further improve its performance. Second, current experiments only used the simple MSE loss as the content term. Future work will investigate other optimization objectives, such as the maximizing likelihood,^[^ ^43^ ^]^ the VGG perceptual loss,^[^ ^76^, ^77^ ^]^ and the adversarial loss.^[^ ^78^ ^]^ Specifically, maximizing likelihood has been demonstrated useful to avoid overestimation of kurtosis values, guarantee data consistency, and improve generalizability. Third, it is unexplored whether additional images with other contrasts that are often acquired along with diffusion MRI data, e.g., high‐resolution T_1_‐weighted data, can further improve DIMOND accuracy. Finally, only in vivo data from healthy subjects were used in this study. Future work will evaluate the performance of DIMOND on patient data.

## 6 Conclusion

This study proposes a fast and self‐supervised diffusion model parameter estimation method entitled DIMOND that can be universally used for fitting any model. For fitting the widely adopted diffusion tensor model, DIMOND outperformed OLS regression and achieved 17.3%, 9.6%, 0.6%, and 3.8% lower MSE for FA, AD, MD, and V1 on HCP‐1Shell data. The NN trained on HCP data generalized well to CDMD data and could be further fine‐tuned to achieve results more similar to those from subject‐specific training. DIMOND also successfully worked for more sophisticated kurtosis model and the microstructural model in NODDI. For DKI, DIMOND outperformed OLS regression, IWLS regression, and CWLS regression, achieving 15.9% to 92.4%, 15.9% to 92.2%, and 1.6% to 39.0% lower MAEs for various DKI metrics on HCP‐2Shell data. Coupled with the CWLS loss, DIMOND removed physiological outliers and noise from resultant parameter estimates, achieving up to 5.0% further reduced MAEs. For NODDI, DIMOND outperformed grid search gradient descent and Brute–Force approach, achieving 1.5% to 11.6%, and 4.2% to 17.3% lower MAEs for various NODDI metrics on HCP‐3Shell data, respectively. For the most time consuming NODDI modeling fitting, DIMOND only needed 24.1 min for subject‐specific training, 32 s for directly applying a pre‐trained NN, and 7.9 min for fine‐tuning and applying a pre‐trained NN, much shorter than 12.3 and 2.3 h required by NODDI‐Toolbox and Dmipy. To summarize, DIMOND is universally effective for any diffusion model, self‐supervised to train, fast to run, easy to implement, deploy and distribute, making it a next‐generation tool to simplify and accelerate diffusion MRI analysis for a wider range of applications in neuroscientific studies and clinical practice.

## Conflict of Interest

The authors declare no conflict of interest.

## Author Contributions

Z.L. conceptualized the idea for the study; designed methodology; developed software; performed formal analysis; wrote the original draft; wrote, reviewed, and edited the final manuscript. Z.L. conceptualized the idea for the study; designed methodology; wrote, reviewed, and edited the final manuscript. B.B. conceptualized the idea for the study; designed methodology; wrote, reviewed, and edited the final manuscript. H.‐H.L. conceptualized the idea for the study. K.Y. conceptualized the idea for the study and acquired resources. S.H. conceptualized the idea for the study. H.L. conceptualized the idea for the study and acquired resources. Q.T. conceptualized the idea for the study; designed methodology; acquired resources; wrote the original draft; wrote, reviewed, and edited the final manuscript; performed data curation; and supervision.

## Supporting information

Supporting information

## Data Availability

The data that support the findings of this study are available from the corresponding author upon reasonable request.
