# Supplementary material for: DIMOND: DIffusion Model OptimizatioN with Deep Learning
Source: Adv Sci (Weinh). 2024 Apr 18;11(24):2307965. doi: 10.1002/advs.202307965 (PMC11200022; doi:10.1002/advs.202307965)
Supplement: Supplementary file 1 — Supporting information [file ADVS-11-2307965-s001.pdf]

ADVANCED SCIENCE
Open Access

Supporting Information

for *Adv. Sci.*, DOI 10.1002/advs.202307965

DIMOND: DIffusion Model OptimizatioN with Deep Learning

*Zihan Li, Ziyu Li, Berkin Bilgic, Hong-Hsi Lee, Kui Ying, Susie Y. Huang, Hongen Liao and Qiyuan Tian**

Supporting Information

DIMOND: DIffusion Model OptimizatioN with Deep learning

*Zihan Li, Ziyu Li, Berkin Bilgic, Hong-Hsi Lee, Kui Ying, Susie Y. Huang, Hongen Liao, and Qiyuan Tian**

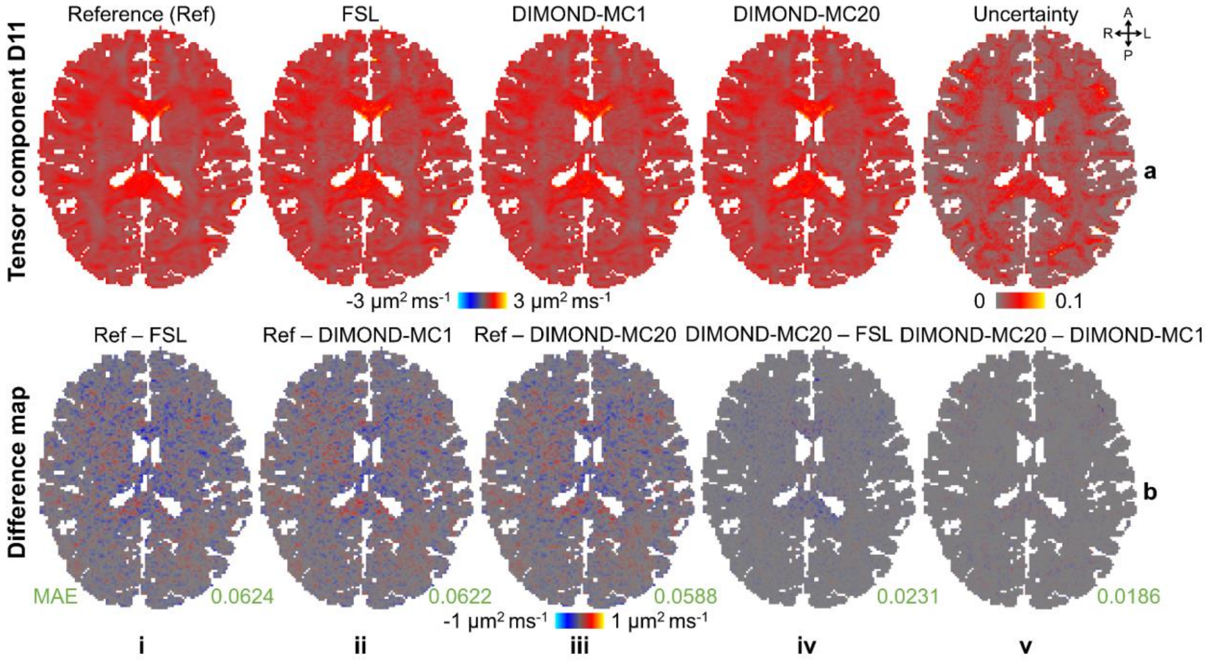

Figure S1. Tensor component D_{11} from HCP-1Shell data. a) Exemplary axial image slices of the tensor component D_{11} generated from all available data using FSL (reference, a(i)) and those generated from sub-sampled data using FSL (a(ii)) and DIMOND (a(iii) and a(iv)), as well as the uncertainty map measured from the 20 inferences (a(v)) are shown. b) The difference maps between reference and FSL results from sub-sampled data (b(i)), reference and DIMOND results (b(ii) and b(iii)), DIMOND-MC20 and FSL result (b(iv)), and DIMOND-MC20 and DIMOND-MC1 result (b(v)) are shown. The mean absolute errors (MAE, green) are listed to quantify the tensor component similarity.

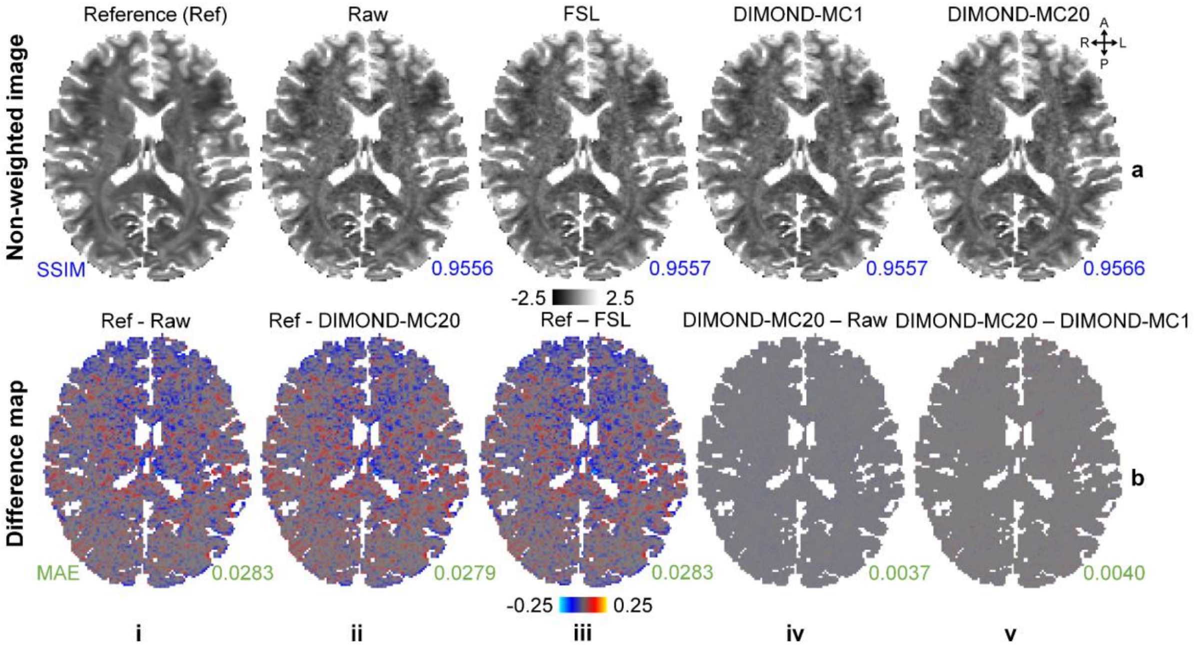

Figure S2. S_0 image synthesized using tensor model. a) Exemplary axial image slices of b = 0 image (S_0) volumes from reference data (a(i)), raw acquired data (a(ii)), and those synthesized from tensors generated using FSL (a(iii)) and DIMOND (a(iv) and a(v)) from sub-sampled data of a representative HCP subject are shown. b) Difference maps between reference and raw acquired image (b(i)) reference and DIMOND-MC20 result (b(ii)), reference and FSL result (b(iii)), DIMOND-MC20 result and raw acquired image (b(iv)), and DIMOND-MC20 result and DIMOND-MC1 result (b(v)) are shown. The structural similarity indices (SSIM, blue) to quantify the similarity between displayed image volumes and the reference and mean absolute error (MAE, green) of difference maps are listed.

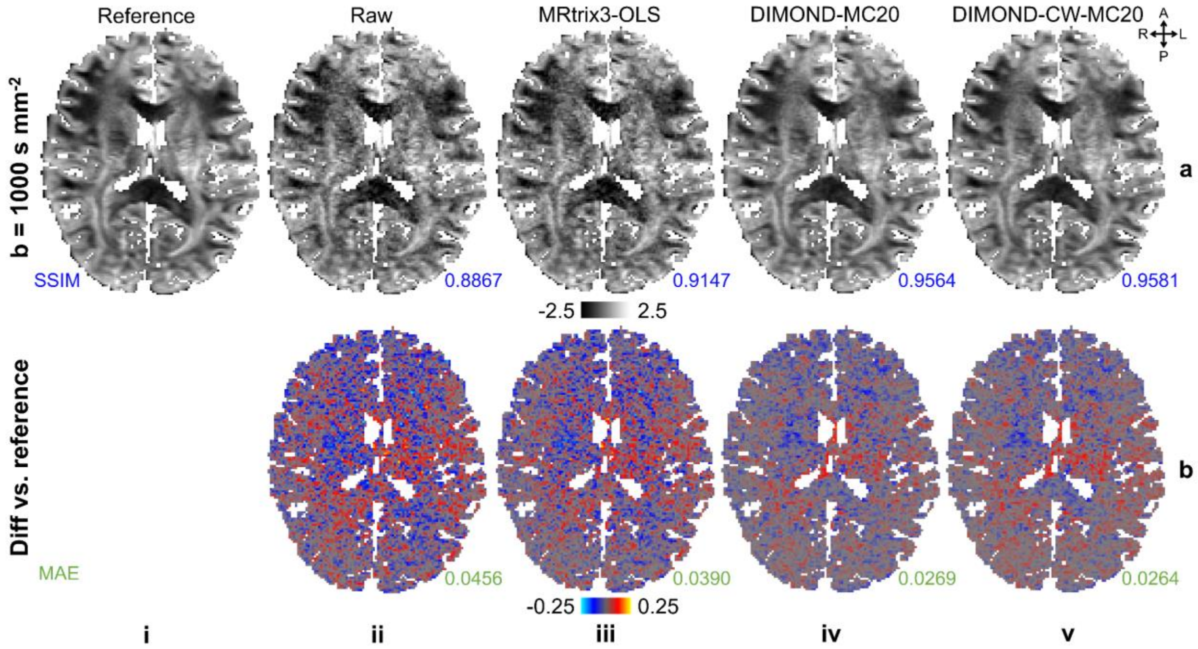

Figure S3. DWI of low b-value synthesized using kurtosis model. a) Exemplary axial image slices of diffusion-weighted image (DWI) volumes (b = 1000 s mm^{-2} along [0.94, 0.28, 0.19]) from the reference data (a(i), synthesized using reference kurtoses via the kurtosis model), raw acquired data (a(ii)), and those synthesized from kurtoses generated using MRtrix3-OLS (a(iii)), DIMOND-MC20 (a(iv)) and DIMOND-CW-MC20 (a(v)) from sub-sampled data of a representative HCP subject are shown. b) Difference maps between the reference and resultant DWIs are displayed, with structural similarity indices (SSIM, blue) and mean absolute errors (MAE, green) listed to quantify the difference.
